# The impact of pituitary adenomas on cognitive performance: a systematic review

**DOI:** 10.3389/fendo.2025.1534635

**Published:** 2025-04-30

**Authors:** Ting-Chia Young, Kai-Yen Lin, Wan-Cheng Li, Chi-Ning Huang, Wen-Hsuan Tsai

**Affiliations:** ^1^ Department of Medicine, Mackay Medical College, New Taipei, Taiwan; ^2^ Department of Medical Education, Taipei Veterans General Hospital, Taipei, Taiwan; ^3^ Division of Endocrinology and Metabolism, Department of Internal Medicine, Mackay Memorial Hospital, Taipei, Taiwan

**Keywords:** pituitary tumor, cognitive performance, neuropsychological abnormalities, transsphenoidal, radiotherapy

## Abstract

**Purpose:**

Increasing evidence suggests that beyond classical endocrine and visual symptoms, patients with pituitary adenoma (PA) may experience neurocognitive impairment, potentially resulting in reduced productivity and diminished quality of life. Prior studies have used diverse cognitive assessment tools across heterogeneous populations, leading to inconsistent findings. To address the variability, our study systematically analyzes the assessment batteries used in previous research, clarifying their corresponding cognitive domains. We seek to provide a more consistent and comprehensive understanding of the neurocognitive implications associated with PAs.

**Methods:**

This study adhered to the Preferred Reporting Items for Systematic Reviews and Meta-analyses reporting guideline. Individual patient-level data, including clinical characteristics, tumor subtype, treatment interventions, hormonal status, and psychological outcomes, were systematically collected. Cognitive assessment tools were categorized according to their corresponding cognitive domains to facilitate domain-specific analyses.

**Results:**

This systematic review included 70 studies encompassing a total of 3,842 patients with PA. Of these, 60 studies employed either objective neuropsychological tests or subjective questionnaires to evaluate cognitive function. The most frequently utilized assessment was the Digit Span test, with 42.9% of studies reporting significant impairments in complex attention and executive functioning among patients with PA. Twelve studies focused on structural brain changes as assessed by magnetic resonance imaging, with half documenting volumetric reductions in gray matter. Across the various PA subtypes, a consistent decline in discrete cognitive domains was observed, most notably in memory and executive function. Treatment-related data were provided in 59 studies. Perioperative changes in cognitive performance were described in 14 studies, of which 11 reported post-surgical improvement in at least one cognitive domain. Twenty studies investigated the potential adverse effects of radiotherapy on cognitive function; among them, 16 found no significant differences following treatment. Eight studies examined the association between tumor size and cognitive impairment; seven reported no statistically significant correlation. In contrast, 24 studies identified a significant relationship between hormonal dysregulation and cognitive decline.

**Conclusions:**

The literature contains heterogeneous findings about the cognitive performance, nature of cognitive impairment, and subsequent effects of treatment. Patients with PA may experience cognitive decline in specific areas and are notably affected by hormone levels, while treatment may lead to cognitive recovery. The proposed tiered cognitive evaluation approach can improve assessment consistency in future practice.

## 1 Introduction

Pituitary adenomas (PAs), comprising 10-15% of all intracranial tumors, represent the most common tumor type within the pituitary gland (1). Symptoms can vary among individuals with PA and are largely dependent on factors such as tumor type and size. These symptoms can manifest as mild conditions such as acromegaly due to excess growth hormone (GH) secretion or Cushing’s disease, resulting from elevated levels of adrenocorticotropic hormone (ACTH). More severe consequences can include hemianopia resulting from a mass effect or even pituitary apoplexy (2).

While the clinical features of PA populations are highly heterogeneous, there is a growing body of evidence suggesting neurocognitive impairment in patients with PA (3–5). PAs may elevate the risk of impaired cognitive function at an earlier age and further lead to mood disturbances, decreased productivity, and compromised quality of life (QoL) (6, 7). The mass effect of PAs, referring to the physical compression of surrounding brain tissue, may result in increased intracranial pressure, displacement of adjacent neuroanatomical structures, and other maladaptive changes, particularly when the tumor extends into the suprasellar region. (8, 9). In addition to these structural consequences, disruptions in hypothalamic and pituitary hormonal axes have also been implicated in neurocognitive dysfunction. Such hormonal disturbances appear to be more pronounced in functional pituitary adenomas (FPAs) than in nonfunctional PA (NFPAs) (10, 11). However, it remains unclear whether cognitive impairment in patients with PA is primarily attributable to the tumor’s mass effect or to endocrine dysregulation. To date, no consensus has been reached regarding a unified pathophysiological mechanism underlying PA-related cognitive dysfunction.

Transsphenoidal pituitary surgery (TSS) is the first-line therapy for all PAs requiring surgical resection, except for prolactin (PRL)-secreting adenomas (5, 12). Despite the overall safety and efficacy of TSS, certain studies have reported the risk of neurocognitive impairment following treatment, while others have observed cognitive improvement postoperatively (4, 13, 14). Radiation therapy (RT), including gamma-knife radiosurgery (GKRS), may be used as a salvage option in adjuvant and recurrent settings (15). Although RT for PAs has a low rate of permanent side effects, toxicities, such as hypopituitarism and cranial nerve injury, are still observed (16). These complications have the potential to place patients at a higher risk of treatment-related cognitive dysfunction (17).

Previous studies that have examined the influence of PAs on neurocognition typically used objective cognitive measures, such as the Mini-Mental State Examination (MMSE), Montreal Cognitive Assessment (MoCA), and Cambridge Cognitive Examination (CAMCOG), or domain-specific tests targeting particular cognitive functions, such as subsets from the Wechsler Adult Intelligence Scale (WAIS) (18–20). Due to the substantial diversity in cognitive assessment methods and the variability in the population studies, the effect of PAs on cognitive function remains insufficiently defined in the existing literature.

This review aimed to provide an update on the neurocognitive implications in patients with PA. Our study collected various cognitive measurements and identified shared perspectives on PA-related neurocognitive impairment across different cognitive domains, tumor subtypes, and treatment approaches.

## 2 Method

### 2.1 Protocol

This systematic review was conducted according to the Preferred Reporting Items for Systematic Reviews and Meta-Analyses (PRISMA) guidelines (21).

### 2.2 Search strategy

We systematically searched the PubMed and Embase libraries up to April 6, 2024 (the date of the last search) to identify relevant articles reporting neurocognitive impairments in PAs. The complete search strategy combined various search terms for PA (e.g., pituitary neoplasm, pituitary tumor, and pituitary cancer) and cognitive function (e.g., cognitive, neurocognitive, and cognitive functions). The detailed search elements are available in **Supplementary Table 1**.

### 2.3 Study selection

After removing duplicates, all titles and abstracts were screened independently by four researchers in a blinded manner (TCY, KYL, WZL, and CNH), and any disagreements were resolved through discussion and overseen by the senior author (WHT). Potentially relevant articles were selected for full-text screening. The eligibility criteria for study characteristics were based on the PICOS acronym.

- Population: Patients with PA
- Phenomenon of interest: Patients with PA and treatment
- Comparator: Patients with PA but without treatment
- Outcomes: Cognitive change
- Study design: Both retrospective observational studies and prospective longitudinal studies were included.

Several exclusion criteria were employed as follows:

- Case reports and reviews
- Studies that solely focus on neuropsychological issues without addressing cognition-related outcomes.
- Studies have included patients with hormone imbalances, not solely resulting from PAs, such as Cushing’s syndrome.

All retrieved full texts were further assessed by four authors, and any disagreements were resolved through discussion and consensus, or by consultation with the senior reviewer.

### 2.4 Data extraction

A standardized data collection form was prepared to extract relevant information from the included full texts, including the characteristics of the study (authors, publication year, country of origin, sample size, and study design), characteristics of participants (age, sex, inclusion/exclusion criteria, PA subtypes, hormone status, and tumor size), characteristics of the intervention (type of surgery or radiotherapy, time elapsed between surgery and cognitive testing, and follow-up time), and characteristics of the outcome (cognitive tests implemented and results of each cognitive test).

### 2.5 Quality evaluation

To evaluate the quality of the included studies, the risk of bias was independently assessed by two authors (WZL and CNH). Bias in cognitive assessment is often controversial and frequently debated (22); therefore, in the “selection bias” domain, we conducted analyses on age, gender, and intellectual ability. We also evaluated the “detection bias” due to the relatively subjective nature of different cognitive function tests. Furthermore, special consideration was given to assessing “analysis bias,” as cognitive function cannot be directly measured but rather inferred by examining performance on carefully designed tests (23). The judgement for each entity was classified as “low risk”, “high risk”, or “unclear”.

### 2.6 Common cognitive measures

Frequently used cognitive assessment tools in the included studies and their corresponding cognitive domains are listed in **Table 1**.

**Table 1:** Breakdown of the frequently used cognitive assessment tools in the included studies, detailing the employed methods for evaluating different cognitive domains.

Measurement	Measured cognitive domains
DGS	Forward: verbal short-term memory, attention
	Backward: working memory
TMT	Part A: processing speed
	Part B: executive function
AVLT	Verbal short-term memory and long-term memory
SCWT	Selective attention, processing speed, and inhibit cognitive interference
RFFT	Executive function, cognitive flexibility
LMW test	Verbal memory, working memory, attention, processing speed
WMS	Logical memory, verbal paired associates, visual reproduction, brief cognitive status exam, designs, spatial addition, and symbol span
DSST	Motor speed, attention, and visuo-perceptual function
RCFT	Visuospatial abilities, memory, attention, planning, and working memory
MMSE	General cognitive impairment
Spatial Span	Visuospatial working memory
Block design	Visuospatial abilities
DDT	Visual neglect, response inhibition, and motor perseveration
LDST	Processing speed, working memory, attention, and cognitive flexibility
COWAT	Verbal fluency
D2 test	Attention
RMTF	Visual reproduction
CAMCOG	General cognitive function
MoCA	General cognitive function
Corsi	Visuospatial working memory
WCST	Cognitive flexibility
ERPs	Inhibitory control

DGS, Digit Span; TMT, Trail Making Test; AVLT, Rey Auditory Verbal Learning Test; SCWT, Stroop Color and Word Test; WMS, Wechsler Memory Test; RFFT, Ruff Figural Fluency Test; LMW, Luria’s Memory Words; DSST, Digit Symbol Substitution Test; RCFT, Rey Complex Figure Test; MMSE, Mini-Mental State Examination; LDST, Letter-Digit Substitution Test; COWAT, Controlled Oral Word Association Test; RMTF: Recognition Memory Test for Faces; CAMCOG, Cambridge Cognition Examination; MoCA, Montreal Cognitive Assessment; Corsi, Corsi Block-Tapping Test; WCST, Wisconsin Card Sorting Test; ERP, Event-Related Potential; DDT, Digit Deletion Test.

In Digit Span (DGS) of WAIS, subjects are presented with a sequence of numbers and are asked to repeat them back in the same order (forward DGS) or in reverse order (backward DGS). The forward subset measures the short-term memory and attention while the backward subset measures executive function, especially working memory (24–26).

The digit symbol substitution test (DSST) from the WAIS is a paper-and-pencil test presented on a single sheet of paper that requires a subject to match symbols to numbers according to a key located on the top of the page. To perform well on the DSST, subjects require intact motor speed, attention, and visuoperceptual functions (27).

Trail Making Test (TMT) is divided into part A and part B. In part A, subjects connect numbered circles in ascending order while they connect circles alternating between numbers and letters in part B. Part A focuses on processing speed and part B targets executive function (28).

Wechsler Memory Scale - Fourth Edition (WMS-IV) is a comprehensive test measuring various aspects of memory. It contains seven subtests: logical memory, verbal paired associates, visual reproduction, brief cognitive status exam, designs, spatial addition, and symbol span. Logical memory and visual reproduction further separated into immediate and delayed conditions, which are administered 20–30 minutes apart (24).

The Stroop Color and Word Test (SCWT) is designed to evaluate various cognitive domains, including selective attention, processing speed, and inhibit cognitive interference (29). Subjects are required to read three different tables as fast as possible. Two of them represent the “congruous condition” in which participants are required to read names of colors (henceforth referred to as color- words) printed in black ink and name different color patches. Conversely, in the third table, named color-word condition, color-words are printed in an inconsistent color ink (for instance the word “red” is printed in green ink). Thus, in this incongruent condition, participants are required to name the color of the ink instead of reading the word (30).

The Rey auditory verbal learning test (AVLT) consists of three different scores describing immediate memory (short-term memory score, total memory score, learning score) and one score for delayed memory (31). Five presentations of a 15-word list are given to subjects, each followed by attempted recall. This is followed by a second 15-word interference list (list B), followed by recall of list A. Delayed recall of list A was measured 30 min after the immediate recall (32, 33).

The Controlled Oral Word Association Test (COWAT) assesses phonemic verbal fluency by instructing participants to produce as many unique words as possible beginning with specified letters (e.g., F, A, S) within timed intervals. The number of correct responses reflects both lexical retrieval efficiency and executive functioning, including the ability to initiate responses and inhibit repetitions or rule-violating word forms (34).

The Ruff Figural Fluency Test (RFFT) evaluates nonverbal executive function by requiring participants to generate novel geometric designs by connecting dots within five timed matrices. Performance is scored based on the number of unique figures and errors, providing insight into cognitive flexibility, planning capacity, and inhibition control (161).

The Wisconsin Card Sorting Test (WCST) measures executive function, cognitive flexibility and abstract reasoning. Participants sort cards according to unstated, changing rules involving color, shape, or number. Feedback guides performance adaptation. Outcomes include the number of categories achieved and perseverative errors, reflecting the ability to shift cognitive strategies in response to feedback (162).

The D2 Test of Attention measures selective and sustained attention using a cancellation task. Participants cross out target letters with specific markings under time constraints. Total processed items, omissions, and errors are recorded. Results reflect visual scanning speed, attentional accuracy, and concentration capacity under controlled time pressure (163).

Rey Complex Figure Test (RCFT) assesses visuospatial construction and memory. Participants are instructed to copy a complex geometric figure and reproduce it from memory after short and long delays. Scoring includes 18 figure components. Results provide insight into planning, organization, and both immediate and delayed visual memory performance (164).

The Digit Deletion Test (DDT) evaluates sustained attention and visual discrimination. Participants review rows of digits and are instructed to cross out all instances of a designated target digit. The number of correct deletions and commission errors serves as an index of attentional control and error monitoring (165).

Spatial Span, an additional subtest of the WMS-IV, measures visuospatial working memory. Participants repeat sequences of tapped blocks in the same (forward) or reverse (backward) order. The task continues until failure on two sequences of the same length. Outcomes reflect short-term memory capacity and spatial attention (166).

The Block Design subtest of WAIS assesses visuospatial reasoning and problem-solving. Participants arrange colored blocks to replicate increasingly complex geometric patterns within a time limit. Performance is evaluated by accuracy and completion time, offering a measure of visual-motor integration, spatial perception, and executive planning (167).

Electroencephalography (EEG) with event-related potentials (ERPs) has been widely used for the assessment of inhibitory response of executive function (35). In Go/Nogo paradigm, which is most frequently used in our included study, participants view visual stimuli of single (Nogo) or double triangles (Go) on a central computer screen in a semidark room. Participants pressed a button quickly for Go stimuli but not for Nogo stimuli. Two major ERP-related components from Go/Nogo tasks assess inhibitory control (36). The N2nogo, appearing at 200-300 ms, shows increased negative deflection at frontocentral electrodes for Nogo versus Go stimuli, indicating conflict detection (37). The P3nogo, at 300-600 ms, shows a greater positive peak at anterior electrodes for Nogo tasks, reflecting conflict inhibition. Difference waves (N2nogo minus N2go, or P3nogo minus P3go) specifically indicate frontal inhibitory control function (38).

Aside from ERPs, Low-Resolution Electromagnetic Tomography (LORETA) can also be applied to identify the origins of EEG signals recorded from the scalp (39). This technique, which creates 3- dimensional distributions to represent EEG sources, offers a linear solution to the inverse problem of EEG source localization (40).LORETA uses a model of the head divided into three spherical shells, representing the scalp, skull, and brain compartments.

## 3 Results

### 3.1 Study selection

**Figure 1:**
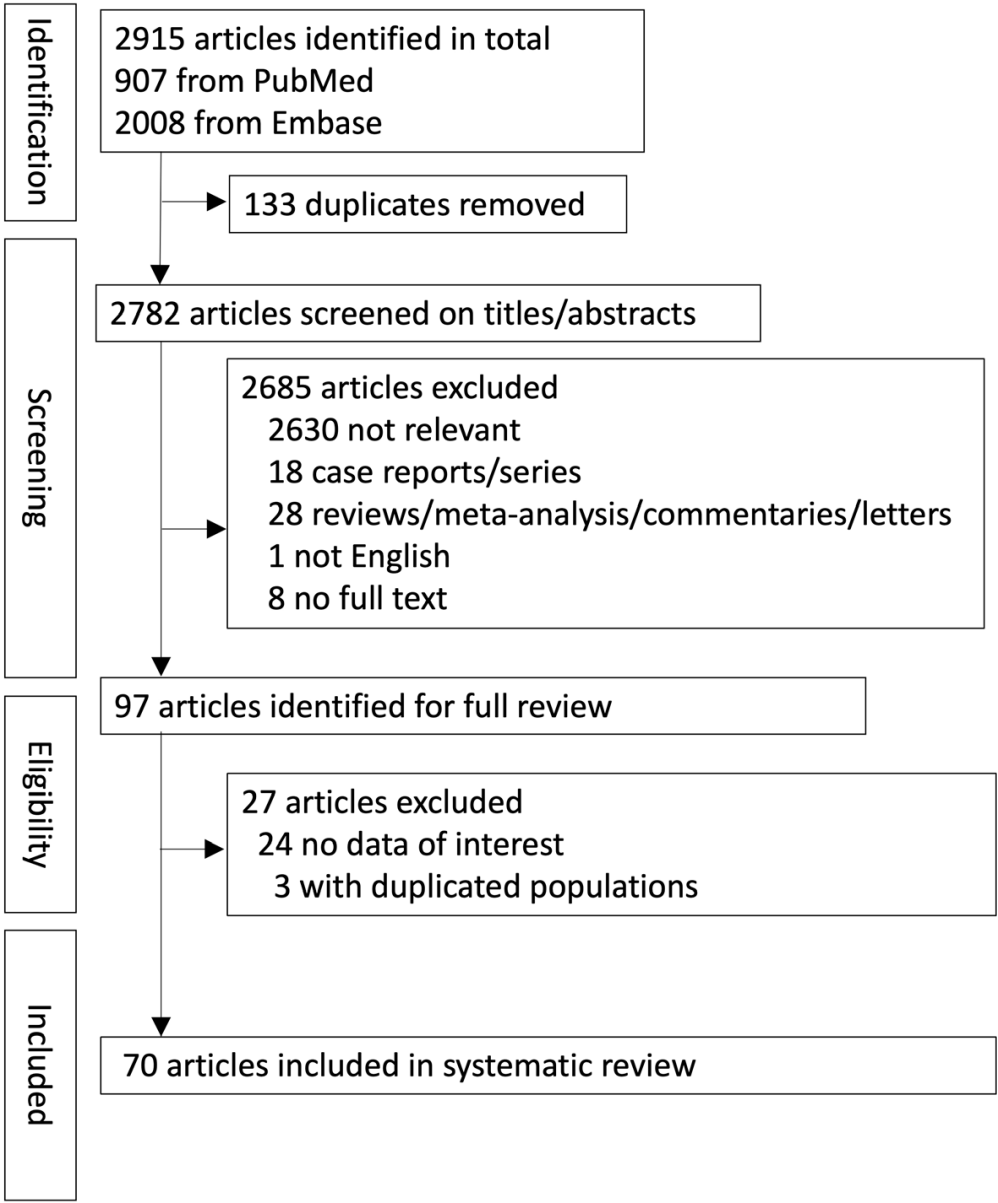
PRISMA flow diagram demonstrating inclusion of the studies.

In total, 2915 records were retrieved from the databases (PubMed, 907; Embase, 2008). A summary of the study screening process and reasons for exclusion is provided in the PRISMA flowchart (**Figure 1**). Seventy papers met all the inclusion criteria for the systematic review. There were 57 retrospective (n = 57/70, 81.4%) and 13 prospective studies (n = 13/70, 18.6%).

### 3.2 Study characteristics

**Figure 2:**
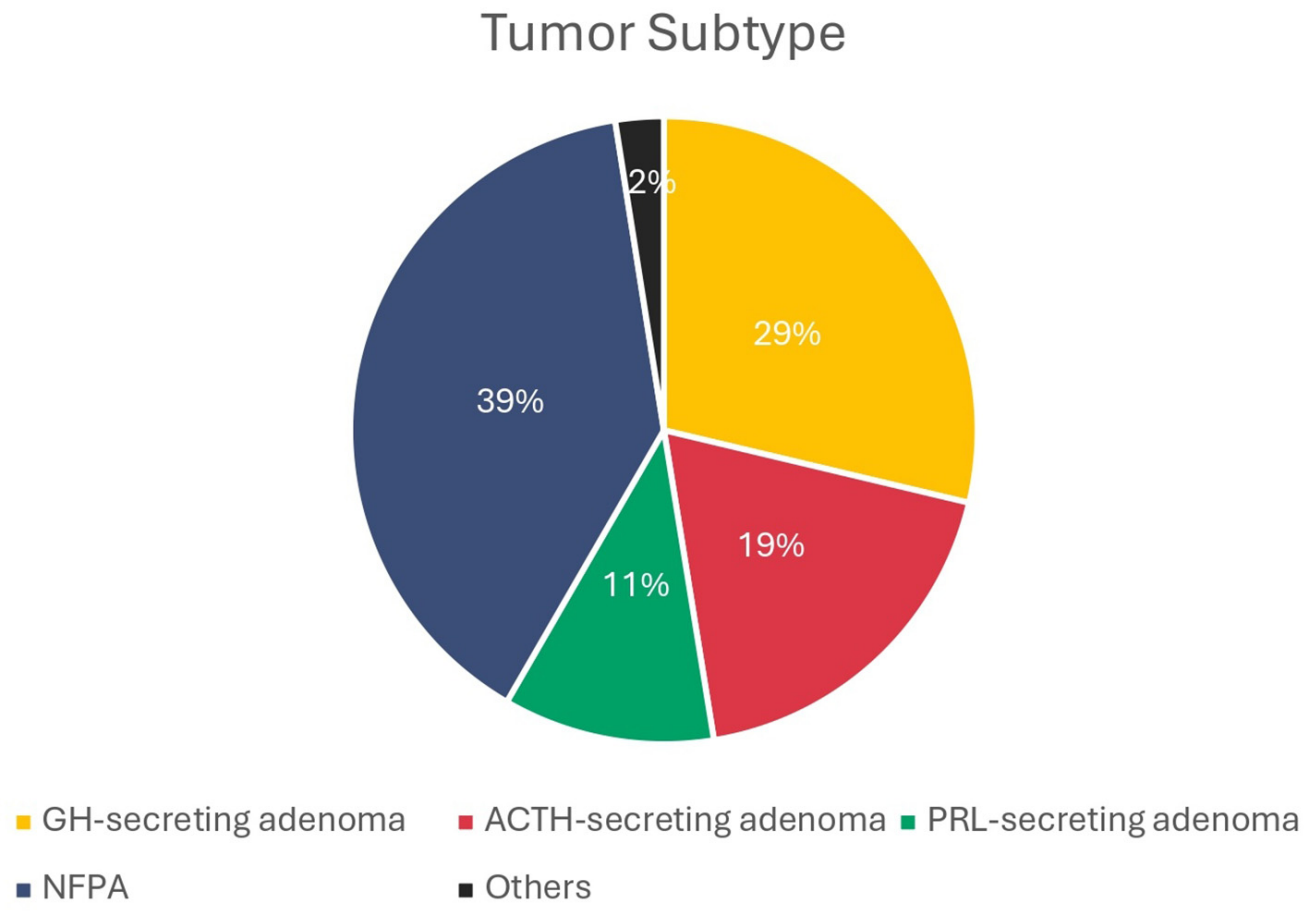
Percentage of tumor subtypes (patients with unspecified subtype are excluded). GH, growth hormone; ACTH, adrenocorticotropic hormone; PRL, prolactin; NFPA, non-functional pituitary adenoma.

The characteristics of the included studies are presented in **Supplementary Table 2**. Of the total 3842 patients diagnosed with PA, 3448 patients were specified with a particular tumor type (89.7%). NFPA accounted for the highest frequency (39.2%), followed by GH-secreting adenomas (28.7%), ACTH-secreting adenomas (18.7%), and PRL-secreting adenomas (10.9%) (**Figure 2**).

### 3.3 Study quality

Details of the risk of bias assessment in the included studies are presented in **Supplementary Table 3**. Most of the included studies had a low risk of selection bias, with a good match between patients with PA and healthy controls in terms of age, sex, and educational background. Analysis bias was predominantly evident, with some studies relying on a single measure from a single task to draw conclusions. Furthermore, most studies had a low risk of detection bias because they used previously validated cognitive assessment tools to evaluate cognitive function.

### 3.4 Cognitive measures

#### 3.4.1 Cognitive assessment tool

**Figure 3:**
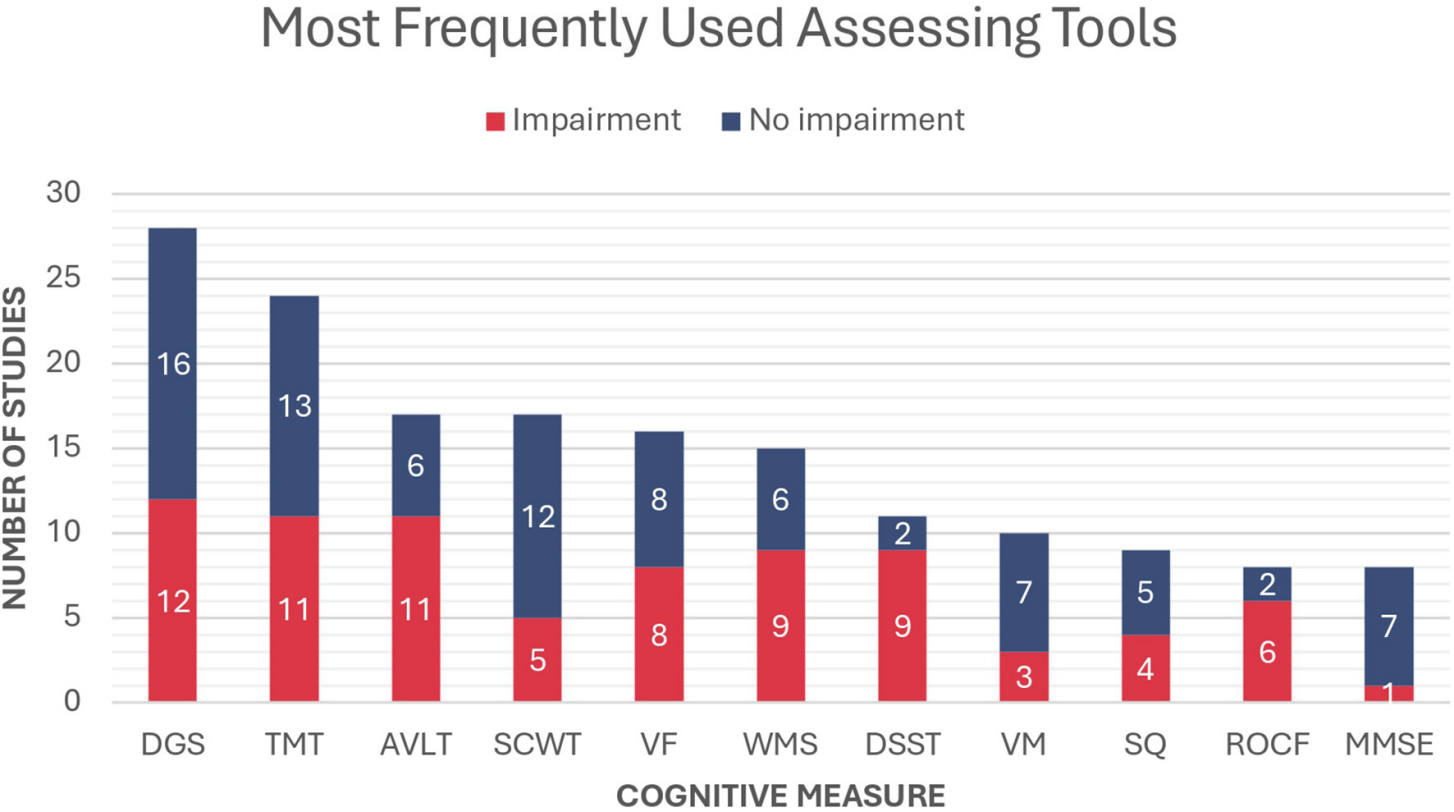
Most frequently used cognitive measures in the included studies and the proportion of reported impairment in patients with PA compared to the normal population. DGS, Digit Span; TMT, Trail Making Test; AVLT, Rey Auditory Verbal Learning Test; SCWT, Stroop Color and Word Test; VF, Verbal Fluency; WMS, Wechsler Memory Test; DSST, Digit Symbol Substitution Test; VM, Verbal Memory; SQ, Self-questionnaire; ROCF, Rey-Osterrieth Complex Figure; MMSE, Mini-Mental State Examination.

Sixty studies (60/70, 85.7%) used either objective or subjective questionnaires to measure the cognitive levels of the participants. **Figure 3** provides an overview of the most commonly used cognitive assessment tools. The DGS emerged as the most frequently used test, reported in 28 studies. Among these, 12 studies documented a significant decrease in scores among patients with PA compared with the normal population. The TMT was the second most frequently used assessment tool, reported in 24 studies. This was followed by the AVLT, which evaluates verbal memory and was utilized in 17 studies. Both assessments demonstrated poorer performance among patients with PA compared with healthy controls, as reported in 11 studies for the TMT and 11 studies for the AVLT.

#### 3.4.2 Magnetic resonance imaging

In the reviewed papers, there were 12 studies (12/70, 17.1%) reporting structural changes detected by MRI as the primary outcome (**Table 2**). Among these, three studies documented decreased gray matter volume, primarily in the temporal region. Another three studies found increased gray matter volume, affecting the hippocampus, cerebellum, or total brain volume. Two studies reported brain volume gains following successful surgical treatment. Additionally, one study identified microbleeds, radionecrosis, and cerebral edema in patients who received radiotherapy. Significant correlations between brain structure and cognitive performance were observed in five studies, while three studies found no such associations. Two studies examined brain function in patients with PA. One reported hypoactivation of the ventromedial prefrontal cortex, while the other observed increased resting-state functional connectivity between the limbic network and the subgenual subregion of the anterior cingulate cortex.

**Table 2:** Structural changes detected by MRI in PA patients compared to the normal population, including findings on gray matter volume, functional connectivity, and post-treatment effects.

MRI findings	Studies (n)	Percentage (%)
Decreased gray matter volume	3	25.0
Increased gray matter volume	3	25.0
Brain volume gain after surgery	2	16.7
Significant correlation between brain structure and cognition	5	45.5
No correlation between brain structure and cognition	3	25.0
Hypoactivation in the ventromedial prefrontal cortex	1	8.3
Increased resting-state functional connectivity	1	8.3
Microbleeds, radionecrosis, cerebral edema (post-radiotherapy)	1	8.3

#### 3.4.3 Electroencephalography

EEG was employed in 10 studies (10/70, 14.3%), of which 8 specifically investigated ERPs to evaluate cognitive and neural processing (**Table 3**). The visual Go/No-go paradigm was the most frequently used task, applied in 4 studies; among these, 2 reported significantly prolonged reaction times in patients with PA. The P300 component was the most commonly examined ERP marker, with 5 studies reporting reduced amplitudes and 1 study noting delayed latencies. One study further demonstrated postoperative improvement in P300 amplitudes. Findings related to P200 and N200 components were more variable: increased P200 amplitudes were observed in 1 study and decreased amplitudes in another; N200 amplitudes were reduced in 3 studies and elevated in 1. Two studies employed subtraction methods to analyze Go vs No-go trials, with 1 reporting enhanced N2d amplitudes in PA patients and the other detecting no significant changes before and after surgery. Less commonly used paradigms included emotional stimuli (2 studies), modified flanker tasks (1 study), and auditory oddball paradigms (1 study). Additionally, LORETA analysis was conducted in 2 studies, both of which revealed decreased activity in the parahippocampus, dorsolateral prefrontal cortex, inferior frontal cortex, and prefrontal cortex.

**Table 3:** Characteristics of studies assessing cognition with electrocardiography and result of PA patients compared to normal population.

First author (year)	Electrodes Setup	Paradigm	Components	Result
Tanriverdi (2009) (78)	Fz, Cz, Pz, Oz referred	Auditory order paradige	P300 amplitudes and latencies	Lower P300 amplitudes
Lean-Carrion (2010) (46)	The international 10–20 system	Not applicable	Low-resolution brain electromagnetic tomography	Decreased activity in the parahippocampus, the dorsal lateral prefrontal region, inferior frontal lobe and prefrontal cortex
Martín-Rodríguez (2013) (141)	The international 10–10 system	Not applicable	Low-resolution brain electromagnetic tomography	Decreased activity in the parahippocampus, the dorsal lateral prefrontal region, inferior frontal lobe and prefrontal cortex
Cao (2017) (151)	Fz, FCz, Cz, referred to the left mastoid	Visual Go/Nogo paradigm	N200 amplitudes and latencies, P300 amplitudes and latencies	Lower Nogo-N200, Nogo-P300, N2d and P3d
Song (2018) (168)	64-channel cap, refereed to the nose site	Deviant–standard–reverse	N170 amplitudes and latencies, P250 amplitudes and latencies	Reduced expression-related mismatch negatively response to sad faces
Song (2020) (51)	64-channel cap, refereed to both mastoids	Visual Go/Nogo paradigm	P300 amplitudes and latencies	Lower P300 amplitudes with improvement after surgery Delay P300 latencies
Cao (2021) (52)	64-channel cap, refereed to both ear lobes	Visual Go/Nogo paradigm	N200 amplitudes and latencies, P300 amplitudes and latencies	Increased reaction time Lower P300 amplitudes in both Go and No-go conditions Lower N200 No-go amplitudes
Cao (2020) (53)	Fz, F3, F4; FCz, FC3, FC4; Cz, C3, C4, referred to the left mastoid	Visual Go/Nogo paradigm	N200 amplitudes and latencies, P300 amplitudes and latencies	Lower P300 amplitudes in both Go and No-go conditions No change in N2d before and after surgery.
Cao (2021) (14)	64-channel cap, refereed to both ear lobes	Emotional stimuli	P200 amplitudes and latencies	Higher P200 amplitudes which decreased after surgery
Chen (2022) (54)	32-channel cap, referred to Cz	Modified flanker paradigm	P200 amplitudes and latencies, N200 amplitudes and latencies, P300 amplitudes and latencies	Increased reaction time Lower P200, P300 and higher N200 amplitudes

### 3.5 Impaired cognitive domains

**Figure 4:**
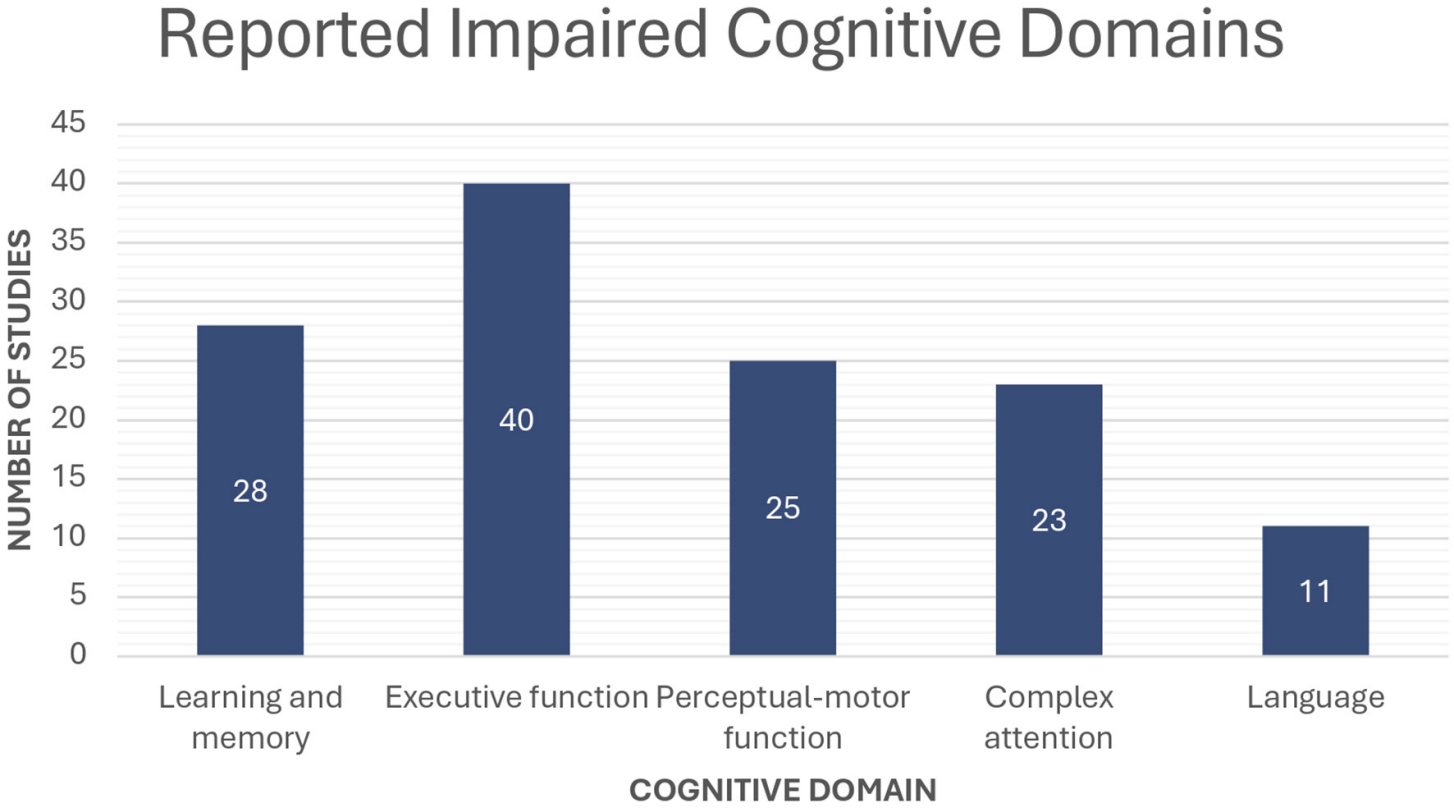
Number of studies reporting cognitive dysfunction in patients with PA across different cognitive domains compared to the normal population.

In all studies analyzed, there was a consistent finding of a significant decline in particular cognitive domains in patients with PA compared with healthy controls (**Figure 4**). Among the analyzed studies, executive function was the most frequently affected domain, with dysfunction reported in 40 out of 70 studies. Within this category, working memory deterioration was identified in 18 studies, indicating deficits in maintaining and manipulating information. Memory impairment was the second most commonly reported domain, documented in 28 studies. Notably, 19 studies specifically identified long-term memory deficits, suggesting impairments in retention and retrieval processes. Perceptual-motor function was reported as impaired in 25 studies, with 17 studies highlighting decreased processing speed, indicating slower cognitive-motor responses. Additionally, 11 studies identified challenges in visuoconstructional reasoning, which may affect spatial awareness and problem-solving abilities. Complex attention deficits were noted in 23 studies, suggesting difficulties in maintaining focus and effectively managing multiple cognitive tasks.

### 3.6 Tumor subtypes

**Table 4** summarizes domain-specific cognitive impairments across different PA subtypes in comparison with the healthy population. Among these, GH-secreting adenoma was investigated in 17 studies, with deficits primarily observed in executive function (n = 13), attention (n = 6), and visual memory (n = 6). Additionally, impairments in short-term memory (n = 5), verbal memory (n = 4), and verbal fluency (n = 4) were also reported. Processing speed deficits (n = 2), perceptual-motor dysfunction (n = 2), and visuospatial impairments (n = 1) were less frequently noted. For PRL-secreting adenoma, 7 studies evaluated cognitive outcomes, with executive function deficits being the most frequently reported impairment (n = 7). Attention deficits (n = 4) and verbal memory impairments (n = 2) were also identified, while processing speed deficits (n = 1), perceptual-motor dysfunction (n = 2), and visuospatial impairments (n = 1) were less commonly reported. In ACTH-secreting adenoma, 9 studies assessed cognitive impairments, with deficits in verbal memory (n = 7), executive function (n = 8), and attention (n = 6) emerging as the most frequently reported dysfunctions. Additionally, short-term memory impairments (n = 6), visual memory deficits (n = 5), and verbal fluency impairments (n = 4) were noted. Long-term memory (n = 1), processing speed (n = 1), perceptual-motor (n = 2), and visuospatial dysfunctions (n = 1) were less frequently observed. For NFPA, 8 studies explored cognitive dysfunction, with executive function impairments (n = 7) and verbal memory deficits (n = 5) being the most commonly reported. Attention impairments (n = 3), short-term memory deficits (n = 2), and processing speed dysfunction (n = 2) were also noted. Less frequent impairments included verbal fluency deficits (n = 1), as well as perceptual-motor (n = 2) and visuospatial impairments (n = 2).

**Table 4:** Summary of cognitive impairments of different PA subtypes compared to healthy controls.

Declined Cognitive Domains Across Different Subtypes	Studies (n)	Percentage (%)
GH-secreting adenoma	17	
• Attention	6	35.3
• Executive Function	13	76.5
• Short-term Memory	5	29.4
• Long-term Memory	1	5.6
• Verbal Memory	4	23.5
• Visual Memory	6	35.3
• Verbal Fluency	4	23.5
• Processing Speed	2	11.8
• Perceptual-motor	2	11.8
• Visuospatial	1	5.6
PRL-secreting adenoma	7	
• Attention	4	57.1
• Executive Function	7	100.0
• Verbal Memory	2	28.6
• Processing Speed	1	14.3
• Perceptual-motor	2	11.8
• Visuospatial	1	5.6
ACTH-secreting adenoma	9	
• Attention	6	66.7
• Executive Function	8	88.9
• Short-term Memory	6	66.7
• Long-term Memory	1	11.1
• Verbal Memory	7	77.8
• Visual Memory	5	55.6
• Verbal Fluency	4	44.4
• Processing Speed	2	22.2
• Perceptual-motor	2	22.2
• Visuospatial	1	11.1
NFPA	8	
• Attention	3	37.5
• Executive Function	7	87.5
• Short-term Memory	2	25.0
• Verbal Memory	5	62.5
• Verbal Fluency	1	12.5
• Processing Speed	2	25.0
• Perceptual-motor	2	25.0
• Visuospatial	2	25.0

Across PA subtypes, two studies found no significant cognitive differences. However, other studies reported varying outcomes depending on the subtype comparisons. Regarding NFPA, two studies observed comparable cognitive outcomes between NFPA and GH-secreting adenoma, while two others found similarities between NFPA and FPA. In contrast, ACTH-secreting adenoma patients demonstrated poorer cognitive performance in multiple aspects. Three studies identified worse verbal memory in ACTH-secreting adenoma patients compared to other PA subtypes, with one study further specifying that these patients had poorer verbal memory and fluency than those with NFPA. Another study found that ACTH-secreting adenoma patients undergoing radiotherapy experienced worse cognitive outcomes than GH-secreting adenoma patients receiving the same treatment. Additionally, one study reported more severe cognitive impairment in ACTH-secreting adenoma patients compared to NFPA. Moreover, one study found that cognitive impairment in ACTH-secreting adenoma) was comparable to that in NFPA. Another study highlighted that treatment effects, rather than PA subtype, played a more critical role in determining cognitive outcomes. GH-secreting adenoma patients exhibited cognitive advantages in certain domains. One study found that they outperformed NFPA patients in processing speed, prospective memory, and semantic memory. Similarly, another study indicated that FPA patients had superior cognitive function compared to NFPA.

### 3.7 Treatment effect

A total of 59 studies (59/70, 84.3%) provided comprehensive details regarding the treatments administered to patients. Out of 3571 patients, 2709 (75.9%) underwent TSS, 171 (4.8%) underwent transfrontal surgery, 40 (1.1%) underwent craniotomy, 784 (22.0%) underwent RT, 555 (20.5%) received medication, and 201 (5.6%) did not receive any treatment. There were 13 prospective longitudinal studies (13/59, 22%) with an average follow-up time of eight months after treatment. Summary of treatment effect on cognition in PA patients is presented in **Table 5**. Cognitive differences before and after surgery were reported in 14 studies. Eleven studies reported postoperative improvement in at least one cognitive domain. The most commonly reported domains for improvement were executive function and memory, reported in six studies. Attention improved in four studies. Three studies compared the cognitive outcomes between surgical and non-surgical patients. One study found no significant difference, while the other two indicated that patients undergoing surgery tended to have worse cognition, particularly in verbal and non-verbal memory. Twenty studies investigated whether additional radiotherapy, including GKRS, negatively affected patients’ cognitive performance. Sixteen studies reported no difference in cognitive performance between patients who received radiotherapy and those who did not.

**Table 5:** Summary of cognitive outcomes in PA patients based on treatment modalities.

Treatment Effect on Cognition	Studies (n)	Percentage (%)
Studies assessing cognitive differences before/after surgery	14	
• Postoperative improvement in at least one domain	11	78.6
• Executive function improved after surgery	6	42.9
• Memory improved after surgery	6	42.9
• Attention improved after surgery	4	28.6
Studies assessing cognitive differences with/without surgery	3	
• Worse outcome in surgery group	2	66.7
• No difference	1	33.3
Studies assessing the impact of radiotherapy on cognition	20	
• No significant effect of radiotherapy on cognition	16	80.0

### 3.8 Hormone level and tumor size

In total, 24 studies (24/70, 34.3%) revealed a significant correlation between hormone levels and the severity of cognitive dysfunction in patients with PA (**Table 6**). Among these, PRL emerged as the most frequently reported hormone in seven studies. These studies revealed a negative correlation between PRL levels and attention (n= 2), memory (n= 2), perceptual-motor function (n= 3), executive function (n= 3). In contrast, one study indicated that improvements in processing speed were more pronounced in patients with baseline PRL levels above the median. GH and IGF-1 levels were reported in six studies and were negatively linked to executive function (n= 2), memory (n= 3), and perceptual-motor function (n= 1). Cortisol levels were investigated in three studies, two of which showed a negative association with memory. The remaining study highlighted that persistent hypocortisolism, rather than hypercortisolism, detrimentally impacts perceptual-motor function and attention.

**Table 6:** Association of hormone levels and tumor size with cognitive impairment in PA patients, summarizing correlated cognitive domains and the number of studies reporting these associations.

Factors and Correlated Cognitive Domains	Studies (n)	Percentage (%)
Prolactin	7	
• Perceptual-Motor Function	3	42.9
• Executive Function	3	42.9
• Attention	2	28.6
• Memory	2	28.6
Growth Hormone & Insulin Like Growth Factor-1	13	
• Perceptual-Motor Function	1	16.7
• Executive Function	2	33.3
• Memory	3	50.0
Cortisol	3	
• Perceptual-Motor Function	1	33.3
• Attention	1	33.3
• Memory	2	66.7
Tumor Size	8	
• No correlation with cognition	7	87.5
Suprasellar Extension	2	
• Perceptual-Motor Function	2	100.0
• Memory	1	50.0
• Attention	1	50.0

Eight studies (8/70, 11.4%) addressed the potential association of tumor size with cognitive impairment (**Table 6**). Among these, seven studies found no significant correlation. Two focused mainly on the suprasellar extension of NFPA. One study revealed that attention and perceptual motor function improvements correlated with the extent of suprasellar tumor removal, while another found improvements in memory and perceptual-motor function after surgical resection.

### 3.9 Psychiatric and quality of life outcome

In addition to assessing cognitive impairment, 49 out of 70 studies (49/70, 70%) included psychiatric outcomes (**Supplementary Table 4**). Among these, 11 studies identified a significantly higher prevalence of depression compared to healthy controls, while 10 studies reported an increased prevalence of anxiety. Other psychiatric conditions, including apathy, irritability, and social phobia, were noted in six studies. Despite these findings, 10 studies reported no association between psychiatric morbidity and cognitive deficits in PA patients. However, one study suggested that anxiety disorders may worsen perceptual-motor function impairment, while another found that both anxiety and depression contribute to long-term memory impairment.

Nine (9/70, 12.9%) studies investigated the QoL of patients with PA (**Supplementary Table 4**). Out of these, five studies reported a significant decrease in QoL among patients with PA compared with healthy controls. Two studies suggested that RT might have the potential to further diminish the quality of life. Additionally, one study indicated an improvement in quality of life after TSS.

## 4 Discussion

### 4.1 Principle findings

This comprehensive review included 70 studies spanning a 50-year period, involving nearly 4,000 patients. Although derived from studies using a wide range of cognitive assessment tools, our data indicate significant cognitive impairment in patients with PA, particularly affecting executive function and memory. Surgical treatment has been associated with improvements in cognitive function, while RT has no detrimental effect on cognitive performance. Tumor size was not associated with the severity of cognitive dysfunction; instead, our findings suggest that hormonal imbalances are the primary factor underlying impaired cognition in patients with PA.

Previously, Pertichetti et al. examined the impact of PAs on cognition and found altered neurocognitive and neuropsychological functions and reduced QoL (41). However, in that study, cognitive dysfunction was examined primarily in relation to tumor subtypes rather than through an analysis of specific cognitive assessment tools. In contrast, our systematic review offers a more granular classification of cognitive function by systematically categorizing impairments into distinct cognitive domains. This refined approach enables a more precise evaluation of cognitive dysfunction in patients with PA and yields clinically relevant insights into the specific domains most affected. On the other hand, studies included in the review by Pereira et al. had focused on psychopathological aspects (42). Other related reviews have also focused on specific tumor subtypes; nevertheless, many original studies have included various tumor subtypes among their subjects and analyzed them all together in a single analysis (43, 44).

### 4.2 Impaired cognitive domains

#### 4.2.1 Executive function

Executive function was the cognitive domain assessed in most of the included studies. The DGS is the most commonly used test in these studies. In 12 of the 28 studies that implemented the DGS, a significant decrease was observed in patients with PA. Interestingly, some studies only found impaired function in the backward subset, whereas the forward subset remained normal (45–48). These findings indicate that in patients with PA, the working memory aspect of executive function is more susceptible to impairment than verbal memory and attention. TMT was the next most frequently used measurement across the included studies. Part A focused on processing speed, whereas Part B targeted executive function. Similar to the digit span test, some studies only showed decreased scores in the TMT Part B (17, 49, 50). These findings further support the notion that executive function, particularly working memory, is vulnerable in patients with PA.

Since the relationship between ERPs and cognitive performance has been recognized, many researchers have used ERPs to investigate the electrophysiological mechanisms underlying cognitive impairment. In particular, the anterior cortex, which plays a central role in executive function, is critical for inhibitory control processes such as the suppression of irrelevant thoughts or actions, self-monitoring, and self-regulation. Several studies included in this review implemented Go/No-go tasks to evaluate inhibitory function in patients with PA. These studies frequently reported abnormalities in N200 and P300 waveforms, reflecting disruptions in both early (conflict detection) and late (response inhibition) stages of cognitive processing (14, 51–54). Collectively, these findings suggest impaired executive function in this population, specifically involving deficits in inhibitory control.

#### 4.2.2 Memory

Memory assessment in patients with PA is particularly challenging because of the inherent complexity of memory and the diverse range of cognitive tests used for its evaluation (55). Some tests focus on the nature of memory, such as episodic and semantic memory (56–58), while others focus on the form of memory presentation, such as verbal and nonverbal memory (59, 60). Additionally, some tests simply categorize memory based on the duration of recall, as seen in tests of short- or long-term memory (59).

Previous studies have demonstrated impaired verbal memory in patients with PA. The AVLT is the third most frequently utilized assessment tool in the reviewed studies, and it is one of the most commonly employed tests for evaluating verbal memory. In 17 studies using the AVLT, 11 reported significantly lower scores for patients with PA. While the majority of studies reported decreased scores in both immediate and delayed memory (13, 45, 61–64), Tiemensma et al. observed decreased scores only in delayed memory among NFPA patients (65), and Crespo et al. found decreased scores exclusively in delayed memory among patients with GH-secreting adenoma (66).

Among the 15 studies that adopted the WMS-IV to assess memory in patients, three studies found impaired function in logical memory. Peace et al. observed impairments in both immediate and delayed conditions, whereas Grattan-Smith et al. identified dysfunction solely in delayed conditions (13, 50). Yao et al. did not specify the conditions under which they observed the impairments (67). Impaired visual reproduction was reported in four studies. Grattan-Smith et al., Mauri et al., and Sievers et al. identified dysfunction in both immediate and delayed conditions, whereas Tiemensma et al. did not specify the conditions under which impairment was observed (47, 50, 68, 69).

#### 4.2.3 Other domains

Although the DSST was adopted in only 11 of the included studies, nine of these studies reported impairment in patients with PA, marking the highest percentage of impairment across all assessment tools. Although the DSST is designed to identify impairment regardless of its nature and origin or to detect changes within a patient rather than provide an absolute diagnosis, past research has consistently shown lower DSST scores in patients with major depressive disorder (70). Due to its brevity and high sensitivity for detecting cognitive dysfunction in patients with PA, future studies should explore whether the DSST could effectively serve as a screening tool for cognitive impairment in this population.

The SCWT, a neuropsychological assessment tool widely used for both experimental and clinical applications, was included in 17 studies. Five studies reported a longer total time and more errors in patients with PA (4, 45, 49, 68, 71). Shan et al. meticulously administered all three subtests of the SCWT (dot, word, and color conditions) to 42 patients with GH-secreting adenoma. They found no differences between patients and healthy controls in the dot test. However, significant differences emerged in the color test, indicating that patients with GH-secreting adenoma exhibited much poorer executive inhibition as task difficulty increased (71).

Verbal fluency involves several complex operations, including finding the correct words, initiating verbal responses, inhibiting responses that do not meet the criteria, correcting incorrect outputs, and shifting attention to a new search. The verbal fluency test sometimes refers to a specific test (72), while at other times it refers to a category of assessment tools, such as COWAT, the Animal Naming Test or the Switching Verbal Fluency Test (73). Tests measuring verbal fluency were performed in 16 of the included studies, with eight of these studies finding impaired function in patients with PA. In a cross-sectional study by Tiemensma et al., the F-A-S test was used to assess verbal fluency in patients with PA. They found that the number of correct responses and the percentage of repeats were significantly lower, whereas the percentage of errors was significantly higher in patients with GH-secreting adenoma than in matched controls. However, this difference was not observed between the patients with NFPA and their matched controls. Interestingly, there was no significant difference between patients with NFPA and those with GH-secreting adenoma (65). A similar result was observed in a study by Zarino et al., in which patients with ACTH-secreting adenoma showed persistently and significantly worse results than controls and NFPA patients in both phonemic and semantic verbal fluency (45).

### 4.3 Impairment mechanism

The original debate regarding the mechanism of PA in cognitive impairment revolved around the long-term effects of excess hormones and the mass effects caused by the tumor itself. Numerous studies have employed multivariate analyses to explore the potential associations between the severity of cognitive impairment and various factors.

#### 4.3.1 Hormone level

##### 4.3.1.1 PRL-secreting adenoma

A prospective study by Montalvo et al. consisted of patients with PRL-secreting adenoma treated with cabergoline, a relatively selective dopamine D2 agonist. Following treatment with PRL-lowering agents, significant improvements were observed in processing speed, working memory, visual learning, reasoning, and problem-solving. An additional correlation analysis revealed that greater prolactin reduction was significantly associated with shorter completion time on the TMT Part A, indicating improved processing speed. However, overall cognitive changes were not significantly correlated with prolactin levels (10).

The cognitive benefits of cabergoline likely involve multiple pathways, including striatal D2 receptor modulation, prefrontal D1/D2 receptor interactions, and PRL reduction through D2 agonism in the tuberoinfundibular pathway (74, 75). Given that D2 receptors in the prefrontal cortex regulate cognitive flexibility and associative learning, their activation may contribute directly to the cognitive improvements observed with cabergoline (76). Montalvo et al. highlighted PRL reduction as a key factor, as cognitive enhancements were more pronounced in patients with higher baseline PRL levels (10). However, the potential interplay between PRL modulation and direct dopaminergic effects on prefrontal circuits warrants further investigation.

PRL has also been found to be associated with structural changes in the brain, which further affect cognitive performance. In a study by Yao et al., elevated PRL levels were linked to a decline in gray matter volume in the left hippocampus. Subsequent analysis revealed that this decline in gray matter volume was correlated with poorer performance in a story recall test (67). This finding is consistent with the crucial role of the hippocampus in memory formation and consolidation (77).

##### 4.3.1.2 GH-secreting adenoma

In the included studies, GH and insulin-like growth factor-1 (IGF-1) are also among the hormones frequently reported to be associated with the severity of cognitive impairment. Shan et al. assessed 46 patients with GH-secreting adenoma and discovered that IGF-1 levels were inversely correlated with semantic memory and working memory (71). Similarly, Tanriverdi et al. found that GH-secreting adenoma patients exhibited significantly reduced P300 amplitude, which reflects impaired executive function, compared to healthy controls and GH-deficient patients (78).

However, the relationship between GH/IGF-1 and cognition appears to be complex, as both hypersecretion and deficiency have been implicated in cognitive dysfunction. Bülow et al. found that postoperative serum IGF-1 levels were positively correlated with processing speed and negatively associated with visuospatial ability (79). Moreover, it is well-established that both childhood-onset or adult-onset GH deficiencies have been linked to attention and memory issues (80, 81). Similar results have been observed in animal models, where adult-onset hypersecretion of GH/IGF-I leads to short-term improvements in neurocognitive function (82).

GH and IGF-1 play crucial roles in normal brain development and function (83). Point mutations in both the IGF-1 and IGF-1R genes have been demonstrated to increase the risk of cognitive development (84). Kalmijn’s clinical trial found that the maintenance of cognitive function hinges largely on the ratio between IGF-1 and IGF binding proteins-3 instead of the absolute level of IGF-1 (85). Together, these findings suggest that abnormal levels of GH and IGF-1 may contribute to cognitive dysfunction.

##### 4.3.1.3 ACTH-secreting adenoma

Glucocorticoid hormones affect glucose utilization in different tissues, including the brain, and experimental studies suggest that they have a direct central action on cerebral metabolism (86, 87). Hou et al., using 18F-fluorodeoxyglucose positron emission tomography in patients with ACTH-secreting adenoma, demonstrated a significant reduction in cerebral glucose metabolism compared to age-matched healthy controls (88). The observed reduction in cerebral glucose metabolism may be attributed to the direct effects of glucocorticoids on neuronal glucose uptake and utilization. Chronic hypercortisolism downregulates glucose transporter type 1 and type 3, which are essential for neuronal glucose uptake, thereby reducing overall cerebral glucose utilization (86). Additionally, glucocorticoids impair astrocytic glucose metabolism, compromising neuronal energy supply and synaptic function, which may contribute to cognitive deficits​ (89).

In addition to metabolic disruptions, excessive glucocorticoid exposure leads to mitochondrial dysfunction and increased oxidative stress, resulting in decreased ATP production and inefficient energy utilization in neurons (90, 91). These energy deficits may contribute to the structural and functional changes observed in the hippocampus, prefrontal cortex, and other regions critical for cognitive function in patients with ACTH-secreting adenoma. Furthermore, glucocorticoid-induced neurovascular dysfunction can lead to impaired cerebral blood flow regulation, further exacerbating metabolic and cognitive deficits​ (92). Meta-analyses of corticosteroid exposure in humans further support the negative impact of glucocorticoids on cognition. Chronic exposure has been associated with impairments in executive function, memory, and processing speed, with deficits appearing dose-dependent, as higher cumulative cortisol exposure correlates with greater cognitive impairment (93).​

Expanding beyond Cushing’s disease, which is caused by ACTH-secreting adenoma, research on Cushing’s syndrome, a disorder resulting from chronic hypercortisolism of either endogenous or exogenous, origin has revealed similar but sometimes more pronounced cognitive impairments, particularly in cases of adrenal-origin hypercortisolism, where ACTH feedback regulation is absent and systemic cortisol levels remain uncontrolled​ (94). Studies have shown that patients with adrenal CS exhibit greater hippocampal atrophy, more severe white matter integrity loss, and more persistent executive function and memory deficits compared to those with ACTH-dependent hypercortisolism, even after biochemical remission (95, 96). Patients with iatrogenic CS also experience Steroid Dementia Syndrome, characterized by persistent deficits in attention, working memory, and verbal fluency, which may not fully resolve even after discontinuation of corticosteroid treatment​ (97). Neuroimaging studies demonstrate that hippocampal atrophy, cortical thinning, and white matter integrity loss contribute to these long-term cognitive sequelae, with functional imaging indicating persistent disruptions in brain network connectivity even after cortisol normalization​ (98).

Interestingly, while cognitive recovery post-treatment is observed in both ACTH-secreting adenoma and CS, the trajectory differs. Patients with ACTH-independent CS tend to show slower or incomplete recovery, possibly due to prolonged systemic cortisol exposure without ACTH feedback modulation​ (98). Conversely, ACTH-secretin patients undergoing transsphenoidal surgery may experience partial improvement in executive function, though residual deficits in memory and processing speed persist​ (94).

#### 4.3.2 Tumor mass effect

Unlike hormone level, most studies investigating the correlation between tumor size and cognitive dysfunction have yielded negative results, further dismissing the possibility of a mass effect as the primary cause of cognitive impairment (48, 50, 62, 69, 71, 99, 100). It is noteworthy that, except for the study conducted by Kan et al., which included 28 patients with NFPA (99), and the remaining studies consisted solely of patients with FPA. Only Wang et al. identified a negative correlation between tumor size and perceptual ability (11).

A larger tumor size reduces the likelihood of achieving complete resection, subsequently impacting prognosis and increasing the risk of cognitive dysfunction (101); nevertheless, the overall recurrence rate after complete resection falls around 10% to 20% within 5 to 10 years in patients with PA, which is relatively low compared with other intracranial malignant tumors (102). Despite the possibility of residual tumors or recurrence, postoperative radiotherapy and medications have demonstrated great efficacy in controlling PA tumors. The likelihood of requiring a second operation is low, let alone disease progression. Another study that included patients with glioblastoma multiforme, astrocytoma, and oligodendroglioma similarly demonstrated that tumor volume was not predictive of cognitive function (103).

#### 4.3.3 Suprasellar extension

According to the preliminary findings of our review, hormone imbalances, rather than tumor size, is a critical factor contributing to cognitive dysfunction. In addition to hypopituitarism occurring postoperatively, hormonal imbalances has seldom been reported in patients with NFPA. This is because patients with NFPA often present with symptoms of mass effect rather than insidiously developing pituitary dysfunction (104, 105). However, cognitive impairment in patients with NFPA has also been reported in several studies (79, 106–109).

In PAs, suprasellar extension occurs when the tumor extends beyond the sella turcica into the sphenoid sinus, cavernous sinuses, or the suprasellar region (110). In a prospective longitudinal study by Psaras et al., more than 80% of patients with NFPA exhibited suprasellar extension, and removing this extension significantly contributed to the enhancement of neurocognitive function (9). Hendrix et al. conducted a prospective matched control study that included ten patients with NFPA. In that study, patients with NFPA with suprasellar extension experienced preoperative cognitive impairments (8). Interestingly, neither study showed a positive correlation between tumor size and the severity of cognitive dysfunction. While larger tumors carry an elevated risk of suprasellar extension, the cognitive impairments identified in these cases are not solely attributed to tumor size. Instead, they are more likely linked to the extension of the tumor into the suprasellar region, as this can lead to the compression of neural structures even after decompression.

### 4.4 Treatment

Seventy-six percent of the patients underwent TSS, while over one-fifth received RT. These findings are consistent with the latest clinical practice guidelines of the Endocrine Society, which recommend TSS as the primary treatment for all PAs requiring intervention, excluding PRL-secreting adenomas, which are typically managed medically. RT is recommended as salvage therapy for patients with PA that recurs after surgery or for those with residual tumors (5, 12).

#### 4.4.1 Surgical treatment

Most studies exploring the potential cognitive impact on surgical patients have yielded positive results. Wang et al. enrolled 76 patients with PA who underwent TSS. Following the surgical intervention, general cognitive screening tests, including the MMSE and CAMCOG, showed significant enhancement. Moreover, post-treatment improvements were observed in language, abstract reasoning, perception, and memory (11). Marsh et al.’s prospective study, which included 56 patients with PA undergoing TSS, revealed postoperative improvements in memory and processing speed (111).

In contrast, a prospective longitudinal study conducted by Butterbrod et al. investigated the perioperative cognitive status in patients with NFPA. The cognitive function of 38 patients was assessed one day before and three months after TSS. Despite postoperative cognitive dysfunction persisting in 63% of patients, there was an overall improvement in cognitive function in 28% of patients, while 28% experienced a decline after surgery (4). The less significant improvement observed in the study by Butterhod et al. could be attributed to the relatively short follow-up period. In a study by Psars et al., cognitive tests were conducted before surgery and at 3 and 12 months postoperatively. Improvement in episodic memory was not evident until the 12-month mark, and domains such as executive function, which had already shown significant improvement, exhibited even greater enhancement after 12 months (9). Similar improvements have been observed in animal models (112).

However, several studies have failed to identify improvements and have even reported worsening cognitive outcomes associated with surgical approaches. Peace et al. discovered that cognitive performance was poorer in patients who underwent surgery than in those who did not (13, 49). Nevertheless, half of the surgical patients in that study underwent surgery using the trans-frontal approach, which is no longer commonly used because of its potential to cause serious iatrogenic damage.

Common complications of TSS, whether employing an endoscopic or microscopic approach, include CSF leakage, syndrome of inappropriate antidiuretic hormone secretion (SIADH), diabetes insipidus, hypopituitarism, bacterial meningitis, and hyponatremia (113, 114). Among the frequently reported complications, hypopituitarism and hyponatremia have been reported to result in cognitive impairment (115, 116). Supporting this, Bülow et al. examined 23 patients with postsurgical hyposomatotropism and found that serum IGF-I levels were significantly correlated with performance on the DSST and negatively associated with the total number of errors on the Austin Maze test, indicating a link between postoperative hormonal dysfunction and cognitive decline (79). Only three other studies have successfully identified associations between improvements in cognition and patient status. Hook et al. discovered that a decrease in cortisol levels was linked to improvements in verbal memory in patients with ACTH-secreting adenoma (117). Psara et al. demonstrated that preoperative hormone deficiency was negatively correlated with improvements in verbal memory (9). In contrast, Butterbrod et al. found that postoperative cognitive performance was not associated with postoperative hormonal status but was consistently predicted by preoperative cognitive performance with notable effect sizes (4).

Our observations underscore that the reduction in certain hormones correlates with enhanced improvement. It is also noteworthy that a majority of studies have overlooked the assessment of postoperative electrolyte levels, which could potentially provide valuable insights into patient outcomes.

#### 4.4.2 Radiotherapy

According to current guidelines, RT is recommended for residual tumor mass following surgery and when medical therapy is unavailable, unsuccessful, or not tolerated (118). However, RT is associated with late toxicities, including hypopituitarism and secondary primary tumors (119, 120). Moreover, growing evidence suggests that radiation exposure to the CNS disrupts diverse cognitive functions, including learning, memory, processing speed, attention, and executive function (121–123).

Mauri et al. were the first to compare exposure to RT in patients with PA and showed no impact on cognitive performance (47). Among the subsequent 20 studies centered on the same topic, only three reported cognitive impairments following RT, particularly in memory, executive function, and attention (17, 69, 124). Tooze et al. and Castinetti et al. examined the cognitive impact of GKRS, with both studies indicating no discernible neurocognitive differences between a GKRS-treated group and participants not subjected to GKRS. However, both Tooze et al. and Castinetti et al. included a limited number of individuals in their exposed groups (125, 126).

The incidence of cognitive dysfunction in patients undergoing RT typically depends on the radiation fraction, total dose, volume, and region covered (127, 128). Furthermore, excluding the hippocampus from RT coverage has been found to preserve better cognitive ability (129). However, Brummelman et al. conducted precise RT dose–volume reconstructions in the brain, enabling a comparison of radiation exposure in radiation-sensitive brain areas, including the hippocampus and prefrontal cortex. The study found no significant differences between the three-, four-, and five-field RT groups and the non-irradiated patient group (106). According to Brummelman et al., while the dose-response relationship is not the major factor influencing cognition in patients with PA receiving RT, it remains a valuable therapeutic approach in the management of recurrent PAs.

### 4.5 Other neuropsychological outcome

Many studies included in this review also used psychiatric tools to assess patients’ mental status as a secondary outcome. However, because our primary focus was on cognitive performance, studies that examined only psychiatric aspects were excluded. Depression and anxiety have been reported to be more prevalent in patients in several studies. Previous studies have demonstrated that lower cognitive ability is generally associated with higher psychological distress and lower psychological well-being (130). Additionally, negative correlations have been identified between psychosocial stress and cognitive function (131–135). However, nearly all the included studies with advanced investigations into the relationship between cognition and mental status in patients with PA yielded negative results. A case-control study by Andela et al. aimed to identify persistent psychological and cognitive dysfunction in patients with long-remission ACTH-secerting adenoma. Although reduced gray matter volume has been observed in multiple brain regions, the increased prevalence of depression and anxiety among patients with PA has not yet been shown to be significantly associated with these structural changes (136). Several other studies have also failed to find a correlation between cognition and psychiatric problems (46, 47, 49, 66, 69, 126, 131, 137, 138).

The stress hormone cortisol is regulated by the hypothalamic-pituitary-adrenal (HPA) axis. Previous research demonstrated a positive correlation between psychosocial stress and cortisol levels (139, 140). Five studies exclusively included patients with ACTH-secreting adenoma, which allowed for a more focused examination of the relationship between psychological distress, cognition, and cortisol levels (47, 136, 137). However, none of these studies showed a specific relationship between psychological distress and cognition, despite identifying a higher rate of depression or anxiety in these patients.

Our study’s findings on the interaction between cognitive performance and psychological status in patients with PA do not align with those of previous studies examining other diseases. However, as mentioned earlier, most of the studies included in our study considered psychological outcomes as secondary and utilized simple measurements, such as the Hospital Anxiety and Depression Scale or the Beck Depression Inventory, instead of implementing more comprehensive assessments. This should be considered when interpreting the results of the present study.

Despite only five included studies reporting QoL, our findings are consistent with those of prior research, suggesting that PA may potentially lead to decreased QoL. Martín-Rodríguez et al. reported significantly higher overall QoL scores after TSS (141). A longitudinal analysis by Castle-Kirszbaum et al. that investigated the QoL of patients with PA undergoing TSS found an initial decline in QoL within the first three weeks. By the end of 6 weeks, the QoL had nearly reverted to its preoperative baseline. Subsequently, the QoL improved compared with the preoperative baseline at 3, 6, and 12 months postoperatively (142).

Some studies not only documented a decline in QoL due to the presence of the tumor but also indicated a decrease in QoL as a result of RT treatment. Patients who underwent RT experienced poorer physical and mental health outcomes than those who underwent surgery alone (108, 143). Decreased QoL following RT has also been observed in other cancers owing to RT-related side effects (144–146). Recently, intensity-modulated RT has been gradually implemented to mitigate these complications and improve overall QoL (147, 148). Nevertheless, it is important to acknowledge that RT often acts as salvage therapy in patients with PA. Thus, RT typically indicates the presence of a residual tumor following extensive surgical resection, suggesting more advanced disease. This factor can also markedly affect QoL.

### 4.6 Cognitive assessment recommendations for future studies

**Figure 5:**
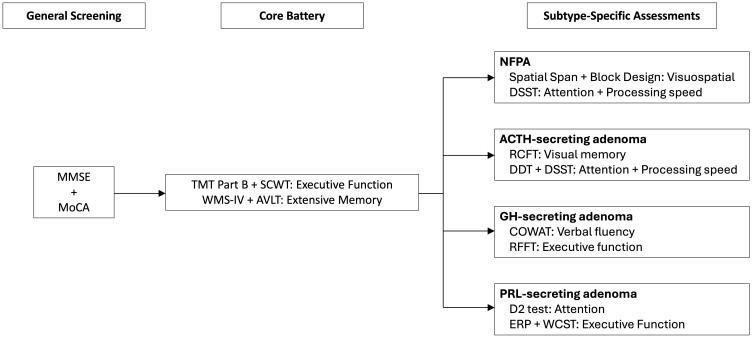
Proposed workflow for tiered cognitive assessment in PA patients for future research and clinical practice. The framework includes overall cognitive screening, core test batteries, and tumor subtype-specific assessments to enhance diagnostic precision and targeted interventions. MMSE, Mini-Mental State Examination; MoCA, Montreal Cognitive Assessment; TMT, Trail Making Test; SCWT, Stroop Color and Word Test; WMS-IV, Wechsler Memory Scale-Fourth Edition; AVLT, Rey Auditory Verbal Learning Test; DSST, Digit Symbol Substitution Test; RCFT, Rey Complex Figure Test; DDT, Digit-deletion Test; COWAT, controlled oral word association test; RFFT, Ruff Figural Fluency Test; ERP, Event-related Potential; WCST, Wisconsin Card Sorting Test.

To establish a more standardized yet adaptable framework for evaluating cognitive function in patients with PA, we propose a tiered cognitive assessment approach that ensures methodological consistency while accommodating the distinct cognitive impairments associated with various PA subtypes (**Figure 5**). These recommendations are derived from the findings of our systematic review, incorporating the most frequently applied cognitive assessment tools in prior studies, thereby enhancing feasibility and ensuring cross-study comparability in future research.

#### 4.6.1 Overall cognitive screening

To ascertain the presence and severity of cognitive impairment in patients with PA, an initial screening using general cognitive assessment tools is recommended. Among the most widely utilized instruments, the MMSE and MoCA provide a broad evaluation of cognitive function. While both tests are effective, studies have demonstrated MoCA’s superior sensitivity in detecting mild cognitive impairment, particularly in assessing deficits in executive function and visuospatial ability, which is especially pertinent to PA patients given the potential impact of hormonal dysregulation on cognitive function (149). In a systematic review comparing the two tools, MoCA showed a significantly higher sensitivity (98%) for detecting mild cognitive impairment compared to MMSE (76%) while maintaining a comparable specificity​ (150). Given the advantages, MoCA is preferable when screening for early cognitive decline, particularly in patients with suspected mild impairment. These preliminary assessments serve as a basis for determining the necessity of a more comprehensive cognitive evaluation.

#### 4.6.2 Core cognitive assessment battery

For PA patients demonstrating cognitive impairment in the initial screening, a comprehensive core test battery is recommended to assess specific cognitive domains commonly declined in PA. Based on our findings, executive function and memory, are the two most frequently reported cognitive impairments in PA patients. Therefore, the core cognitive assessment battery is designed to directly evaluate these domains using well-established and widely applied tests:

- Executive Function: Executive dysfunction is a hallmark cognitive impairment in PA, likely influenced by hormonal imbalances and frontal lobe dysfunction, both of which have been implicated in cognitive control and flexibility deficits (36, 51, 151). Given that executive functions encompass cognitive flexibility, inhibitory control, working memory, and set-shifting, a multimodal assessment approach is essential to capture the full spectrum of impairments. To evaluate cognitive flexibility and set-shifting, the TMT Part B is incorporated. Impairments in TMT-B performance have been consistently linked to frontal lobe dysfunction and have been observed in various neurological and endocrine disorders, underscoring its relevance in PA-related cognitive assessment (28, 152). In addition, inhibitory control and interference processing are assessed using the SCWT. SCWT evaluates the ability to suppress automatic responses and resolve cognitive interference, which is particularly relevant in PA given the potential dysregulation of dopaminergic and corticostriatal pathways associated with inhibitory deficits​ (29). Prior studies have shown that individuals with frontal lobe dysfunction exhibit prolonged reaction times and increased errors in incongruent Stroop conditions, further reinforcing its utility in PA-related cognitive assessments (153, 154). Furthermore, working memory, an essential component of executive function, is assessed through the DGS. Recent findings suggest that DGS is a superior measure of working memory capacity, as it involves both information retention and mental reorganization, thus placing a greater demand on executive control processes​ (155). Given that working memory is integral to higher-order cognition, including problem-solving and decision-making, its assessment provides critical insights into potential deficits affecting daily functioning in PA patients.
- Memory: Given the high prevalence of verbal and visual memory impairments in patients with PA, a comprehensive evaluation is crucial for capturing deficits across multiple memory domains. The WMS-IV provides an extensive assessment of verbal and visual memory, thus allowing for a nuanced analysis of encoding, retention, and retrieval processes. Prior research has demonstrated that WMS-IV is particularly effective in detecting cognitive dysfunction across various neurological populations, with its subtests showing strong sensitivity to memory impairments associated with structural and functional brain abnormalities (156). Moreover, given that memory performance is often influenced by executive function and attentional control, the integration of multiple subtests within WMS-IV enables a more comprehensive cognitive profile, distinguishing true memory impairments from performance variability and ensuring greater clinical accuracy (157). The AVLT is included due to its well-documented sensitivity in assessing verbal memory encoding, consolidation, and delayed recall. AVLT is particularly useful in differentiating pathological memory dysfunction from normal aging, as well as in detecting subtle cognitive deficits linked to neurological disorders. Recent research has further expanded its utility to functional MRI studies, where AVLT paradigms have been employed to examine verbal memory lateralization and hippocampal activation patterns (158). Additionally, demographically corrected normative data for AVLT have been validated across diverse populations, enhancing the reliability and clinical interpretability of its results (159).

#### 4.6.3 Subtype-specific assessments

Given that different PA subtypes have distinct cognitive impairment profiles, additional tests may be incorporated based on specific deficits identified in each subgroup. To build up a more targeted assessment framework, we identified the most frequently reported cognitive impairments in each PA subtype based on our systematic review. This process involved three key steps: (A) determining the cognitive domains most affected in each PA subtype based on the frequency of reports across studies, (B) selecting validated neuropsychological tests that correspond to these impaired domains to ensure robust assessment, and (C) complementing this approach by examining which specific cognitive tests were most frequently reported as impaired across all included studies for each subtype. This bidirectional analysis allowed us to align domain-specific deficits with the most sensitive and commonly utilized assessment tools, thereby enhancing the methodological consistency and clinical applicability of our recommendations.

- GH-secreting Adenomas: Patients showed significant impairments in executive function and verbal fluency. Given the central role of executive dysfunction in this subtype, RFFT is recommended to assess cognitive flexibility. Additionally, verbal fluency deficits are evaluated using COWAT, which provides insights into phonemic fluency and lexical retrieval. These assessments collectively allow for a more precise characterization of cognitive dysfunction in GH-secreting adenomas.
- PRL-secreting Adenomas: The predominant cognitive deficits include executive function and attention. To assess attentional control, the D2 Test is incorporated, as it offers a sensitive measure of sustained and selective attention, which is often impaired in PRL-related cognitive dysfunction. Given the high frequency of executive function impairment reported, WCST and ERPs paradigm are included. The extensive use of ERPs in prior studies ensures their reliability and enhances its applicability as a standardized assessment tool for PRL-related cognitive dysfunction (51, 53, 54). They provide objective electrophysiological measures of cognitive processing, offering insights into the temporal dynamics of executive control and attentional regulation.
- ACTH-secreting adenomas: Impairments in executive function, visual memory, and attention are among the most frequently reported cognitive deficits. Given the relevance of visual memory impairments in this subtype, RCFT is recommended to assess visuospatial memory, organization, and recall performance, which may be affected by ACTH-related neuroendocrine changes. Additionally, attentional deficits and slowed processing speed, as documented in a subset of studies, warrant the inclusion of DDT and DSST, both of which provide objective measures of attentional control and cognitive processing speed. These assessments enable a more refined approach to detecting neurocognitive dysfunction in this population.
- NFPA: Cognitive impairments are frequently reported in executive function and verbal memory, with additional studies noting deficits in processing speed and visuospatial abilities. Given the proportion of studies highlighting visuospatial dysfunction, the Spatial Span and Block Design subtests are incorporated to evaluate visuospatial working memory and constructional ability. Furthermore, the DSST is employed to assess processing speed and attentional control, aligning with prior findings that have identified these domains as vulnerable in NFPA-related cognitive impairment. These targeted assessments enhance the precision of neurocognitive profiling in NFPA, ensuring that domain-specific impairments are systematically evaluated.

This tiered assessment approach ensures that the core cognitive assessment battery captures the most commonly affected domains, while subtype-specific assessments provide additional insights into cognitive impairments unique to each PA subtype, thereby enhancing the precision and clinical relevance of cognitive evaluations in PA research.

## 5 Limitations

This study had some limitations. First, the cognitive assessment tools used in the included studies are highly heterogeneous. Many tests are polyfactorial measures that simultaneously evaluate subdomains pertaining to multiple domains. For example, WCST was designed to measure abstract reasoning. Abstract reasoning does not fit any of the traditional cognitive subdomains outlined in DSM-5 (160). Instead, it includes a multitude of tasks closely associated with executive functions, complex attention, and memory. Moreover, certain domains such as executive function and memory tend to be more readily accessed and contain a greater number of subdomains, often accompanied by a wider array of tests to assess them. Consequently, impairments in these cognitive domains may be reported more frequently. Thirdly, in terms of the investigation of treatment impact on cognition, while our review included several prospective longitudinal studies, some cross-sectional studies merely compared cognitive differences between a pre-treatment group and a post-treatment group from two distinct populations, leading to significant confounding factors. Finally, some of the included studies enrolled participants with multiple subtypes of PA in a single cohort and did not stratify them into subgroups during analysis. This may have led to a reduction in the observed differences in cognitive dysfunction in response to different hormones.

## 6 Conclusions

The literature contains heterogeneous findings regarding cognitive performance, the nature of cognitive impairment, and the subsequent effects of treatment. Patients with PA have been shown to experience cognitive deterioration in certain domains, in which hormonal imbalances may play an important role. The following treatments may be beneficial for cognitive improvement. While cognitive assessment needs to be further standardized, physicians should consider testing cognitive performance in patients with PA and hormonal imbalances. We propose a tiered cognitive evaluation approach, incorporating overall screening, a core test battery, and subtype-specific assessments. This framework in future clinical practice may improve the monitoring, and management of cognitive deficits in PA patients.

## Data Availability

The original contributions presented in the study are included in the article/**Supplementary Material**. Further inquiries can be directed to the corresponding author.
