# Supplementary material for: The impact of pituitary adenomas on cognitive performance: a systematic review
Source: Front Endocrinol (Lausanne). 2025 Apr 30;16:1534635. doi: 10.3389/fendo.2025.1534635 (PMC12074915; doi:10.3389/fendo.2025.1534635)
Supplement: Supplementary file 1 [file Table1.docx]

**Supplement 1.** Search strings used for literature retrieval in PubMed and Embase, along with the number of articles obtained from each database. The searches were conducted up to April 6, 2024, using Boolean operators and Medical Subject Headings terms related to pituitary adenomas and cognitive function.

| Database | Search string | No. of articles |
| --- | --- | --- |
| PubMed | ((Pituitary Neoplasms) OR (Neoplasm, Pituitary) OR (Neoplasms, Pituitary) OR (Pituitary Neoplasm) OR (Pituitary Tumors) OR (Pituitary Tumor) OR (Tumor, Pituitary) OR (Tumors, Pituitary) OR (Pituitary Carcinoma) OR (Carcinoma, Pituitary) OR (Carcinomas, Pituitary) OR (Pituitary Carcinomas) OR (Cancer of Pituitary) OR (Pituitary Cancers) OR (Cancer of the Pituitary) OR (Pituitary Cancer) OR (Cancer, Pituitary) OR (Cancers, Pituitary) OR (Pituitary Adenoma) OR (Adenoma, Pituitary) OR (Adenomas, Pituitary) OR (Pituitary Adenomas) OR (Prolactinomas) OR (Prolactin-Producing Pituitary Adenoma) OR (Pituitary Adenoma, Prolactin-Producing) OR (Pituitary Adenomas, Prolactin-Producing) OR (Prolactin Producing Pituitary Adenoma) OR (Prolactin-Producing Pituitary Adenomas) OR (Prolactin-Secreting Pituitary Adenoma) OR (Prolactin Secreting Pituitary Adenoma) OR (Prolactinoma, Familial) OR (Lactotroph Adenoma) OR (Adenoma, Lactotroph) OR (Adenomas, Lactotroph) OR (Lactotroph Adenomas) OR (Pituitary Adenoma, Prolactin-Secreting) OR (Pituitary Adenoma, Prolactin Secreting) OR (Pituitary Adenomas, Prolactin-Secreting) OR (Prolactin-Secreting Pituitary Adenomas) OR (PRL-Secreting Pituitary Adenoma) OR (Pituitary Adenoma, PRL-Secreting) OR (Pituitary Adenomas, PRL-Secreting) OR (PRL Secreting Pituitary Adenoma) OR (PRL-Secreting Pituitary Adenomas) OR (Adenoma, Prolactin-Secreting, Pituitary) OR (Macroprolactinoma) OR (Macroprolactinomas) OR (Microprolactinoma) OR (Microprolactinomas) OR (Pituitary ACTH Hypersecretion) OR (Hypersecretion, Pituitary ACTH) OR (Cushing's Disease) OR (Cushing Disease) OR (Disease, Cushing) OR (Cushing Disease, Pituitary) OR (Pituitary Cushing Disease) OR (Pituitary Cushing Syndrome) OR (Cushing Syndrome, Pituitary) OR (Pituitary-Dependant Cushing Syndrome) OR (Cushing Syndrome, Pituitary-Dependant) OR (Pituitary Dependant Cushing Syndrome) OR (Pituitary-Dependant Hypercortisolism) OR (Hypercortisolism, Pituitary-Dependant) OR (Pituitary Dependant Hypercortisolism) OR (Pituitary-Dependant Hypercortisolisms) OR (Pituitary-Dependant Hypercortisolism Disorder) OR (Hypercortisolism Disorder, Pituitary-Dependant) OR (Pituitary Dependant Hypercortisolism Disorder) OR (Pituitary-Dependant Hypercortisolism Disorders) OR (Inappropriate ACTH Secretion Syndrome) OR (Adrenocorticotropic Hormone, Inappropriate Secretion) OR (Inappropriate Adrenocorticotropic Hormone Secretion) OR (Acromegaly) OR (Inappropriate Growth Hormone Secretion Syndrome (Acromegaly)) OR (Somatotropin Hypersecretion Syndrome (Acromegaly)) OR (Hypersecretion Syndrome, Somatotropin (Acromegaly)) OR (Hypersecretion Syndromes, Somatotropin (Acromegaly)) OR (Somatotropin Hypersecretion Syndromes (Acromegaly)) OR (Syndrome, Somatotropin Hypersecretion (Acromegaly)) OR (Syndromes, Somatotropin Hypersecretion (Acromegaly)) OR (Inappropriate GH Secretion Syndrome (Acromegaly))) AND ((Cognition) OR (Cognitions) OR (Cognitive Function) OR (Cognitive Functions) OR (Function, Cognitive) OR (Functions, Cognitive) OR (Cognitive) OR (Neurocognitive) OR (Neurocognitive Disorders) OR (Disorder, Neurocognitive) OR (Disorders, Neurocognitive) OR (Neurocognitive Disorder) OR (Organic Brain Syndrome, Nonpsychotic) OR (Nonpsychotic Organic Brain Syndrome) OR (Delirium, Dementia, Amnestic, Cognitive Disorders) OR (Mental Disorders, Organic) OR (Disorders, Organic Mental) OR (Mental Disorder, Organic) OR (Organic Mental Disorder) OR (Organic Mental Disorders) OR (Kandinsky Syndrome) OR (Clerambault Syndrome) OR (Psychoses, Traumatic) OR (Traumatic Psychoses) OR (Mild Neurocognitive Disorder) OR (Disorder, Mild Neurocognitive) OR (Disorders, Mild Neurocognitive) OR (Mild Neurocognitive Disorders) OR (Neurocognitive Disorder, Mild) OR (Neurocognitive Disorders, Mild) OR (Organic Mental Disorders, Psychotic)) | 907 |
| Embase | ('hypophysis adenoma'/exp OR 'hypophysis adenoma' OR 'acromegaly'/exp OR acromegaly OR 'prolactinoma'/exp OR prolactinoma OR 'cushing disease'/exp OR 'cushing disease') AND ('cognition'/exp OR cognition) | 2008 |
