# Supplementary material for: The impact of pituitary adenomas on cognitive performance: a systematic review
Source: Front Endocrinol (Lausanne). 2025 Apr 30;16:1534635. doi: 10.3389/fendo.2025.1534635 (PMC12074915; doi:10.3389/fendo.2025.1534635)
Supplement: Supplementary file 2 [file Table2.docx]

**Supplement 2.** Summary of included studies, detailing study type, participant numbers, tumor types, cognitive measures, objectives, main findings, and conclusions. The table covers retrospective and prospective studies on pituitary adenomas, highlighting affected cognitive domains, statistical significance, and key interpretations for comparison.

| **First author**  **(year)** | **Study type** | **No. of participants**  **and tumor type** | | **Cognition Measures** | **Purposes** | **Main results** | **Conclusion** |
| --- | --- | --- | --- | --- | --- | --- | --- |
|  |  | **PA** | **HC** |  |  |  |  |
| Grattan-Smith (1992) | Retrospective | GHoma: 10  ACTHoma: 13  PRLoma: 15  Chromophobe A: 27 | 21  Chronic illness | ROCFT, COWAT, TMT, WMS, WRMT | To assess the neuropsychological dysfunction in a group of patients irradiated for PAs. | A neuropsychological assessment conducted on 65 patients with PAs revealed impairments in both memory and executive function. | Neuropsychological impairment is common in patients with PAs and represents a significant aspect of their disability that has historically received insufficient attention. |
| Mauri (1993) | Prospective | ACTHoma: 25 | 25 | DGS, Corsi, WMS, RCPM, DSST, Similarities, LCT, TMT, VF, Street’s Completion | To evaluate the cognition of patients with ACTHomas, a comparison was made with HCs. | Patients with ACTHoma demonstrated significant impairments compared to HCs in several cognitive areas: logic memory test (both learning and retrieval), primacy score, DGS backward, visual reproduction (both learning and retrieval), and the DSST. | The results indicate a selective impairment of memory functions, with the number of patients showing significantly impaired mnemonic performance increasing with age. |
| Peace (1997) | Retrospective | 36 | 36 | DGS, AVLT, WMS, RMTF, SWCT, COWAT, BD, TMT | To examine the neuropsychological functioning of a group of patients who had been treated for PAs. | Impairments in memory and executive function were exhibited by the PA patients. These deficits were related to the presence of surgery, but not to the type of surgery. | PA patients do suffer from cognitive dysfunction. The reasons for this are unclear but are likely multifactorial. |
| Guinan (1998) | Retrospective | 90 | 19 | RMT, WMS | To establish the prevalence of cognition impairment in PA patients. | Significant deficits in anterograde memory were observed on WMS-R and RMT across all patient groups compared to healthy controls. No significant differences were found between the individual surgical and radiotherapy treatment groups. | Anterograde memory deficits were observed in all treatment groups compared to HCs. |
| Peace (1998) | Retrospective | NFPA: 22  GHoma: 9  PRLoma: 21  ACTHoma: 10 | 23 | DGS, WMS, RMTF, COWAT, BD, TMT | To examine the neuropsychological status of patients treated for PAs by trans-frontal surgery, TSS , or medical treatment only, with or without radiotherapy. | Comparison of the four groups revealed that nearly half of the trans-frontal, one-third of the TSS, and one-quarter of the non-surgical patients had three or more neuropsychological test scores below the 10th percentile, compared to less than 5% of the controls. | Many patients with treated PAs suffer significant cognitive impairment. |
| Starkman (2001) | Retrospective | ACTHoma: 48 | 38 | WAIS, WMS | To use ACTHoma as a unique human model to elucidate the cognitive deficits resulting from exposure to chronically elevated stress levels of endogenous cortisol. | Scores for four of five verbal IQ subtests were significantly lower in patients with ACTHomas. Their scores were significantly lower for only one nonverbal performance IQ subtest (block design). Verbal learning and delayed recall at 30 minutes were significantly decreased, whereas visual learning and recall were not affected. | Chronically elevated levels of glucocorticoids have deleterious effects on specific cognitive domains, with verbal learning and other verbal functions being more vulnerable than nonverbal functions. The results suggest that both the neocortex and hippocampus are affected. |
| Bülow (2002) | Retrospective | PRLoma: 6  NFPA: 17 | 33 | DGS, BD, CMVM, AM, APT RT-2, APT RT-inhibition | To investigate the incidence of mental disorders and the prevalence of mental distress and cognitive dysfunction in hypopituitary women with untreated GH deficiency compared to HCs. | Hypopituitary women had a higher incidence of mental disorders than HCs. The Global Severity Index from the SCL-90 was higher in patients, with significantly more symptoms of somatization, anxiety, depression, obsession-compulsion, hostility-irritability, and phobic and psychotic symptoms. | Hypopituitary women had a higher incidence of mental disorders, more symptoms of mental distress, and an increased prevalence of cognitive dysfunction. |
| Bellhouse (2003) | Retrospective | PA: 18  Craniopharyngioma: 18 | n/a | WAIS, WRAT | To determine the cognitive and behavioral changes in patients following craniopharyngioma and PA surgery. | Differences were observed in the subjective experience of appetite and the degree of control exercised over eating behaviors. | In the cognitive domains assessed, these two groups have similar outcomes from surgery. |
| Bhansali (2004) | Retrospective | NFPA: 7  GHoma: 4 | n/a | MRI, CT | Describe the clinical spectrum of RIBD in 11 patients who received post-surgery conventional megavoltage irradiation for residual PAs. | The clinical spectrum of RIBD included new-onset visual abnormalities (5 patients), cerebral radionecrosis with altered sensorium (4), generalized seizures (4), cognitive dysfunction (5), dementia (3), and motor deficits (2). | Using an appropriate and well-accepted dose fractionation regimen is crucial in managing PAs to avoid the severe consequences of irradiation. |
| Noad (2004) | Retrospective | NFPA: 33  PRLoma: 9  TSHoma: 1  GHoma: 27  FSHoma: 1 | n/a | RFC, WMS, SCOLP, COWAT, SCWT | To determine if RT for PAs is associated with cognitive impairment and reduced QoL. | Patients with PAs treated with surgery, with or without RT, showed cognitive impairment compared to the normal population. Those receiving RT performed significantly worse on the Stroop test, a measure of executive function, than those receiving only surgery. | Patients treated for PA may experience cognitive impairment, regardless of treatment type. The decline was more pronounced in the RT group, primarily affecting executive function. |
| Heald (2006) | Retrospective | NFPA: 16  ACTHoma: 16 | n/a | CVLT, VOSP, AMIPB, SCWT, TMT, TEA | To investigate cognitive differences between patients treated for ACTHoma and those treated for NFPA. | No significant differences were found in verbal learning, story recall retention, higher executive tasks (SCWT and TMT), or information processing between the groups. | No difference in cognitive function between patients with ACTHoma and NFPA. |
| Hook (2007) | Prospective | ACTHoma: 27 | n/a | BSRT, DGS, VF | To investigate whether cortisol's negative effects on cognition can be reversed following successful treatment. | A specific pattern of significant cognitive and morphological improvement follows successful treatment, with recovery in VF and recall, but not in brief attention. | At least some of the harmful effects of prolonged hypercortisolemia on cognitive function are potentially reversible, up to 18 months post-treatment. |
| van Beek (2007) | Retrospective | NFPA: 81 | n/a | CFQ | To assess the long-term impact of different treatments on QoL and cognitive problems in patients with NFPAs. | Most QoL and cognitive domains showed a similar score in patients who underwent RT when compared with other patients. | Postoperative RT in patients with NFPA is not associated with reduced quality of life or cognition when compared with surgery alone. |
| Müssig (2009) | Prospective | NFPA: 6  PRLoma: 11  GHoma: 14  ACTHoma: 11 | n/a | TMT, AVLT, DGS, MWT | To investigate whether younger age at the time of treatment is linked to better neuropsychological outcomes following successful intervention for PA. | Age at treatment was significantly associated with TMT part B. | Younger age at treatment in adults has a favorable effect on neurocognitive outcomes after PA surgery. |
| Tanriverdi (2008) | Retrospective | GHoma: 18 | 16 | EEG | To investigate the impact of GH deficiency and excess on cognitive performance through P300 ERP. | The mean P300 amplitude in acromegaly patients was significantly lower than in both HCs and GH-deficient patients across all electrode sites. | Severe GH deficiency leads to prolonged P300 latencies, while acromegaly results in reduced P300 amplitudes. |
| Leon-Carrion (2010) | Retrospective | GHoma: 16 | 16 | COWAT, LMW, TMT, SCWT, THS, ROCFT | To investigate if GH/IGF-I excess affects cognitive functions in untreated GHoma patients and if these changes are linked to neurophysiological correlates. | Short- and long-term memory were the most impaired cognitive functions and negatively correlated with GH and IGF-I levels. No link was found between memory impairment and depression. | Results indicate moderate-to-severe memory impairment and reduced neural activity in specific brain regions, evidencing cognitive and neurophysiological deficits. |
| Psaras (2010) | Retrospective | ACTHoma: 24  GHoma: 37 | n/a | d2, DGS, DSST, IST-2000, TMT, | To assess how current disease status affects neurocognitive function and QOL in treated ACTHoma and GHoma patients, and to identify predictors for post-operative neurocognition and QoL. | Impaired QoL was more pronounced than neurocognitive decrease in both pathologies compared to HCs. This finding was independent of the current status of disease. | The current disease status has a minor impact on postoperative outcomes for QoL and neurocognition in both pathologies. This may be due to significantly improved endocrinopathy after treatment, even if a cure is not achieved. |
| Tiemensma (2010) | Retrospective | ACTHoma: 74  NFPA: 54 | 128 | MMSE, WMS, AVLT, ROCFT, TMT, SCWT, LDST, DDT Figure fluency, FAS test, GIT-2 | To assess cognitive functioning  after long-term cure of Cushing’s disease. | Compared with NFPA patients, patients cured from ACTHomas had lower scores on the MMSE, and on the memory quotient of the WMS. | Cognitive function, reflecting memory and executive functions, is impaired in patients despite long-term cure of ACTHoma. |
| Tiemensma (2010) | Retrospective | GHoma:68  NFPA: 68 | 68 | MMSE, WMS, AVLT, ROCFT, TMT, SCWT, LDST, DDT Figure fluency, FAS test, GIT-2 | To assess psychopathology, personality traits, and cognitive function in patients after long-term cure of GHomas. | Cognitive function in patients cured from GHomas did not differ from HCs or patients treated for NFPA. | Patients with long-term GHoma remission exhibit higher rates of psychopathology and maladaptive personality traits but not cognitive dysfunction, compared to matched controls and those treated for NFPA. |
| Brummelman (2011) | Retrospective | PA: 84 | n/a | RFFT, AVLT | To examine cognitive functions in patients previously treated for NFPA with or without RT. | Patients who underwent RT showed no significant differences on cognition when compared to those who received surgery alone. | Patients with NFPA score significantly lower in cognition compared to reference populations, with RT showing no major impact on cognitive function. |
| Müssig (2011) | Retrospective | NFPA: 6  ACTHoma: 10  GHorma: 13  PRLoma: 9 | 38 | AVLT, TMT, DGS, DSST | To determine if cognitive deficits in patients in long-term remission from PA surgery exceed those in other chronically ill patients. | Attention, attentional speed, executive control, and working memory, were significantly reduced in patients with PAs compared with other chronically ill patients. | Patients with successful PA surgery, compared to other chronically ill patients, show an increased risk of cognitive deficits, highlighting the impact of the brain lesion and its treatment on these dysfunctions. |
| Petry (2011) | Retrospective | NFPA: 18  GHoma: 12  PRLoma: 5  ACTHoma: 1 | n/a | MMSE, DGS, WS, famous face | To comparatively assess cognitive function and quality of life in patients with PAs who developed hypopituitarism post-surgery. | Regarding health-related QoL, significant differences were found in social relationships, with lower scores in the RT group. | Irradiated hypopituitary patients experienced impaired social relationships and more severe depression in their QoL. |
| Psaras (2011) | Prospective | GHoma: 29  ACTHoma: 14  PRLoma: 12  NFPA: 39  Sellar cyst: 12 | n/a | IST-2000, d2, TMT, DGS | To investigate whether memory and executive functions improve after surgical treatment in PA patients. | Concentration, working memory, and attentional speed significantly improved within the first 3 months after surgery. However, episodic memory improvement was not observed until 12 months post-surgery. | In patients with PAs, the removal of suprasellar tumor extensions was the key factor in improving neurocognitive function. |
| Brummelman (2012) | Retrospective | GHoma: 50  NFPA: 24 | n/a | RFFT, AVLT | To compare the cognitive functioning of patients treated for GHomas with those having NFPA. | The total patient group scored significantly poorer than the reference population on memory and executive functioning. However, cognitive performance did not significantly differ between GHoma patients with persistent disease, those in remission, and NFPA patients. | There was no association between previous GH excess or its current medical treatment in acromegaly and cognitive functions like memory and executive functioning. |
| Brummelman (2012) | Retrospective | NFPA: 75 | n/a | RFFT, AVLT | To refine the strategy to correlate radiation dose to radiosensitive brain areas with cognitive test performance in PA patients. | Cognitive test performance did not differ between varying radiation doses applied to the hippocampi and prefrontal cortex. | There were no significant differences on cognitive performance between the three-, four-, and five-field RT groups and the non-irradiated patient. |
| Ragnarsson (2012) | Retrospective | ACTHoma : 43 | 55 | ANT | To study cognitive function in patients with ACTHomas in long-term remission. | Patients performed significantly worse in tests of processing speed, auditory attention, working memory, verbal fluency, and reading speed. | The pattern of cognitive and attentional deficits indicates a global involvement of brain function in patients with ACTHomas in long remission. |
| Sievers (2012) | Retrospective | ACTHoma: 55 | 87 | MRI, TAP, d2, AKT, DGS, SPM, RWT, AVLT | To investigate whether cognitive impairments in patients with GHomas exist and if they are associated with structural brain alterations as defined by MRI. | Up to 33.3% of patients had impairments in attention, 24.1% in memory, and 16.7% in executive function. Overall, 67.3% of patients failed to reach the cut-off level in at least one subtest. | GHoma is characterized by pathological endocrine alterations and associated comorbidities, as well as below-normal performance in cognitive tests, independent of biochemical control, treatment regime, and affective disorders. |
| Andela (2013) | Retrospective | ACTHoma : 25 | 25 | MRI, CFQ | To examine structural changes in the brain in patients with ACTHomas in long-term remission. | Patients had smaller grey matter volumes of areas in the anterior cingulate cortex and greater volume of the left posterior lobe of the cerebellum. | Patients with ACTHomas in long-term remission showed specific structural brain abnormalities |
| Martı´n-Rodrı´guez (2013) | Retrospective | GHoma: 40 | n/a | SCWT, TMT, LMW, ROCFT, DGS, LCT, TH | To compare the neurocognitive functions of patients cured by transsphenoidal surgery for GHomas with those of patients with untreated acromegaly. | Both groups of patients scored significantly poorer than healthy controls on memory tests, particularly those assessing visual and verbal recall. Patients with cured ACTHoma did not perform better on cognitive measures than untreated patients. | The effects of chronic exposure to GH/IGF-I hypersecretion could have long-term  effects on brain functions. |
| Yedinak  (2013) | Retrospective | Active GHoma: 10  Controlled GHoma: 17  NFPA: 14 | n/a | FACT-Cog | To assess patient perception of cognitive deficits in active GHomas versus controlled GHomas versus NFPAs. | The highest perceived prevalence and severity of cognitive dysfunction were among NFPA groups, especially in mental agility, verbal recall, and memory. | All groups of patients with adenoma had variety degree of cognition dysfunction. |
| Bas-Hoogendam (2015) | Retrospective | ACTHoma: 21 | 21 | MRI | To investigate brain activation during  emotion processing in patients with long-term remission of ACTHomas. | There was hypoactivation of the ventromedial prefrontal cortex during the processing of facial expressions (vs. scrambled faces), without alterations in amygdala activation. | These functional alterations may underlie the long-term psychological morbidity in patients with ACTHomas after correction of hypercortisolism. |
| Brummelman  (2015) | Retrospective | NFPA: 43 | n/a | MRI, RFFT, AVLT | To compare cognition and brain abnormalities in treated NFPA patients with and without cognitive impairments. | No differences were seen between NFPA patient with or without cognitive dysfunction for white matter lesions, cerebral atrophy, and abnormalities in temporal lobes or hippocampi. | Brain abnormalities on MRI are not more frequently observed in treated NFA patients with cognition impairments. |
| Crespo (2015) | Retrospective | GHoma: 31 | 31 | IGT, AVLT | To evaluate memory and decision-making in GHoma patients and explore their relationship with affective disorders such as anxiety and depressive symptoms. | GHoma patients showed impairments in delayed verbal memory and more anxiety and depressive symptoms than HCs. | Impaired delayed memory and decision-making in GHoma patients are related to anxiety and depressive symptoms. Providing emotional support could improve their cognitive function. |
| Lecumberri (2015) | Retrospective | GHoma: 66  ACTHoma: 33  PRLoma: 8  GHRHoma: 1  NFPA: 1 | n/a | MMSE, BVRT, WCST, BT | To assess the long-term impact of postoperative two-field conventional RT on the neurocognitive functions of adult patients with operated PAs. | Compared to non-RT patients, RT patients performed significantly worse on BVRT, arithmetic problems, and five categories of the WCST. | In adult patients with operated PA, RT was independently associated with an impairment on verbal memory and executive function, when compared to non-RT patients. |
| Tiemensma (2015) | Retrospective | GHoma: 50 | n/a | IPQ-R | To explore the effectiveness of the drawing test and its association with illness perceptions and QoL in patients with long-term remission from GHomas. | Larger drawings indicated more negative consequences, higher emotional representation scores, and more perceived symptoms attributed to acromegaly. They also reflected more impaired quality of life, particularly disease-specific QoL. | There are strong correlations among the drawing test, illness perceptions, and QoL. The drawing test is a novel, easy tool for assessing patient perceptions after long-term remission of acromegaly. |
| van der Werff (2015) | Retrospective | ACTHoma : 24 | 24 | MRI | To examine the alterations in RSFC in patients with long-term remitted ACTHomas. | Patients showed an increased RSFC between the limbic network and the subgenual subregion of the anterior cingulate cortex. | Increased RFSC in patients with long-term remitted ACTHomas are established under the influence of hypercortisolism. |
| Bala  (2016) | Retrospective | PRLoma: 20 | 20 | ROCF, d2, AVLT, BD, VF, DGS, TMT, BVRT | To evaluate cognitive functions in patients with PRLomas | The group of patients had significantly lower scores on verbal memory, nonverbal memory, and attention tests compared with healthy volunteers. Results in memory and visuospatial tests were significantly negatively correlated with the level of PRL. | Greater hyperprolactinemia was associated with a larger decrease in cognitive performance. |
| Alibas (2017) | Retrospective | GHoma: 42 | 44 | AVLT, DGS, COWAT, SCWT, TMT | To compare depressive mood and cognitive function in patients with GHomas and in HCs. | Patients with acromegaly had lower learning, planning, complex attention and inhibitory function scores than the controls. | The present findings show that GHoma negatively affects learning, attention, and planning. |
| Cao  (2017) | Retrospective | PRLoma, GHoma, NFPA: 26 in total | 26 | EEG | To investigate whether PA patients show a brain processing impairment in inhibitory control. | A larger Nogo-N2 and Nogo-P3 was found in the control group compared with the pituitary group. | Data indicate that individuals with PAs exhibit reduced early and late inhibitory processes, suggesting dysfunction in conflict detection and inhibitory control. |
| Hendrix (2017) | Prospective | PA: 10 | 10 | DGS, DSST, Corsi, VLMT, SCWT, TMT, RWT, WAIS | To focus on the early postoperative cognitive trajectory and its correlation with postoperative imaging in PA patients. | The short-term memory deficit disappeared one week after surgery. Perceptual speed recovered within two months after surgical therapy. | Patients with NFPAs that extend suprasellarly may show preoperative neurocognitive impairments that resolve within two months post-surgery. |
| Shan (2017) | Retrospective | GHoma:; 42 | 42 | SCWT, HSCT, N-back test, SART | To explore various aspects of executive function in patients with GHomas and investigate the causes of dysexecutive syndrome in this group. | The patients exhibited worse results on the SCWT, VF, HSCT and N-back test compared to the HCs | GHoma patients showed severe impairments in semantic inhibition, executive processing, working memory, and executive inhibition, and they were aware of some of these deficits. |
| Wang (2017) | Prospective | FPA: 34  NFPA: 42 | 76 | MMSE, CAMCOG | To identify factors contributing to cognitive impairment and explored the impact of surgical treatments under various conditions on PA patients' cognitive functions. | Cognitive functions significantly improved after surgery, but no differences were observed between the two surgical treatments. | Cognitive functions in PA patients are generally impaired before surgery, unrelated to tumor size or eyesight. Endocrine disorders from FPAs may be the primary cause of these impairments. |
| Yao (2018) | Retrospective | PRLoma: 32 | 26 | MRI, WCST, WMS, DGS | To identify brain structure changes in RPLomas and determine their relationship to cognitive performance and clinical characteristics. | Compared to healthy HCs, patients with PRLomas showed reduced gray matter volume in several brain regions: the left hippocampus, left orbitofrontal cortex, right middle frontal cortex, and right inferior frontal cortex. | In patients with PRLomas, specific abnormalities in brain structures are linked to cognitive impairments and hormonal dysfunction. |
| Montalvo (2017) | Prospective | PRLoma: 10 | n/a | MCCB, BACSSC, CFAN, TMT, CPTIP, SS, UMLNS, HVLT-R, BVMT-R, NAB-M | To determine if reducing prolactin levels with cabergoline in hyperprolactinaemia patients improves cognitive tasks. | Cabergoline treatment led to significant improvements in processing speed, working memory, visual learning, and reasoning/problem-solving. | Prolactin may play a role in cognitive processes, but cabergoline could also have procognitive effects independent of prolactin changes. |
| Solomon (2018) | Retrospective | GHoma: 19 | 19 | TMT, SCWT, VF | To evaluate executive function and QoL in GHoma patients compared to HCs. | GHoma patients performed worse on the TMT Part B and produced significantly fewer words in phonemic fluency tests compared to HCs. | GHoma patients display worse executive functioning than healthy controls and have a decreased QoL. |
| Song  (2018) | Retrospective | PRLoma, GHoma, NFPA: 26 in total | 26 | EEG | To investigate the preattentive processing of emotional faces in patients with PAs. | EMMN related to sad faces was smaller in patients, whereas the EMMN related to happy faces remained the same. | The function of negative emotional processing at the preattentive stage of information processing was impaired in PA patients. |
| Tooze (2018) | Retrospective | ACTHoma: 9  NFPA: 5 |  | WAIS, WMS | To evaluate the effects of GKRS on cognitive functioning in patients with PAs. | No neurocognitive differences were found between the GKRS treated group versus participants not treated with GKRS. | We found no evidence that GKRS impairs the neurocognitive functioning of patients with pituitary disease above any impairment caused by the disease itself. |
| Wang (2018) | Prosepctive | NFPA: 402  GHoma: 49  ACTHoma: 37  PRLoma: 21 |  | MMSE, CAMCOG | To investigate the effect of GA in postoperative clinical application | Compared to the placebo group, the GA-treated group showed significant improvements in orientation, language, memory, praxis, abstract thinking, and MMSE scores one-month post-surgery. After six months, abstract thinking scores remained significantly higher. | GA significantly improved the cognitive functions at the early stage after surgery and had a long-termed efficacy on abstract thinking. |
| Butterbrod (2020) | Prospective | NFPA: 45 |  | CNS Vital Signs | To investigate perioperative cognitive status and course in patients with NFPAs. | Impairment rates were significantly higher in patients than in HCs. While group-level performance remained unchanged over time, 28% of individual patients showed cognitive improvement and 28% showed cognitive decline post-surgery. | Cognitive impairment was present before and after TSS in over half of NFPA patients. |
| Crouzeix (2019) | Retrospective | NFPA: 9  GHoma: 8  PRLoma: 2  GHRHoma: 30 | n/a | MoCA | To evaluate the long-term impact of surgery, RT, and pituitary deficiencies on health-related QoL and cognitive function in NFPA patients. | The MoCA score was abnormal in 41% of NFPA patients. Neither the type of surgery nor radiotherapy affected the prevalence of cognitive disabilities. The depression score was higher in RT patients compared to no-RT patients. | Surgery and RT did not adversely affect cognitive functions; however, QoL was reduced in patients who underwent RT. |
| Kan  (2019) | Retrospective | NFPA: 28  PRLoma: 12  GHoma: 9  GHRHoma: 12  TSHoma: 1  ACTHoma: 1  plurinominal: 1 | 69 | WAIS, WMS | To identify whether the cognitive decline was related to level of hormones and age in PA patients. | Several of the cognitive impairments were more frequently observed in untreated PA patients while several significant correlations were found between cognitive domains and hormone levels, such as free T4 and ACTH. | A significant decline in the cognitive performance of patients with PA prior to medical treatment, especially in older patients, which suggests that hormones and age have the ability to interact and aggravate cognitive decline in patients with PA. |
| Wennberg (2019) | Retrospective | GHoma: 67 | n/a | SSRT, DGS, Corsi, ROCFT, SCWT, TAB, TMT, VF | To study the relationship between sleep disturbances and cognitive dysfunction in a group of GHoma patients. | In linear regression models adjusted for age, sex, BMI, disease duration, and disease activity, poorer sleep quality was associated with lower global cognitive z-score. | Poor sleep quality is associated with poorer QoL, and some evidence that it is associated with poorer cognitive function in GHoma patients. |
| Zarino (2019) | Prospective | ACTHoma: 10  NFPA: 10 | n/a | Corsi, ROCFT, AVLT, TMT, SCWT, WCST, RCPM, VF | To investigate whether psychological and neuropsychological impairments in ACTHomas are related to neural network derangement caused by hypercortisolism, tumor mass, or TSS. | Preoperatively and 3–7 days postoperatively, both subgroups exhibited significant neuropsychological disabilities compared to controls, but only the ACTHoma subgroup did not fully recover over time. | QoL and neuropsychological impairments were observed in all patients early on, regardless of hypercortisolism, tumor mass, or successful surgery. Over time, ACTHoma patients showed persistent QoL changes, especially in social activities, indicating a stronger impact and more psychological and neuropsychological comorbidities than NFPA. |
| Cao  (2020) | Retrospective | PRLoma: 21 | 21 | EEG | To examine changes in the ERPs  and neural oscillations in PRLoma patients. | Lower frontal theta power was found in PA patients in both Go and Nogo conditions. | The frontal theta oscillation was highlighted as the electrophysiological markers of the impaired response control in PRLomas. |
| Marsh (2020) | Prospective | NFPA: 17  PRLoma: 20  ACTHoma: 8  GHoma: 23 | 18 | WAIS, WMS, NART, SRMS, FAS test, RMT, SCOLP | To investigate whether pituitary lesions cause cognitive impairments. | Patients with ACTHomas had lower verbal recognition memory pre-treatment but improved at follow-up, partly due to better depression scores. Patients treated with surgery had poorer verbal recognition memory than HCs at all time points. | Verbal memory impairments in the ACTHoma patients may result from increased cortisol, directly or via depression. In the surgical groups, impairments likely predated treatment, possibly due to factors influencing treatment selection rather than the surgery itself. |
| Song (2020) | Retrospective | PRLoma: 40 | 20 | EEG | To identify electrophysiological markers of response activation and inhibition impairment in PRLomas. | The peak latency of P300 was significantly delayed in both pre- and postoperative groups compared to HCs. Preoperative patients had significantly decreased P300 amplitudes in both Go and Nogo conditions. Six months postoperatively, prolactinoma patients showed increased P300 amplitudes during both tasks. | PRLoma patients experience deficits in response activation and inhibition, which can be improved by surgical treatment. |
| Cao (2020) | Prospective | PRLoma: 20  GHoma: 7  NFPA: 3 | 30 | EEG | To identify factors affecting inhibitory control in PA patients. | N2d amplitudes were significantly lower in both presurgery and postsurgery patients compared to the HCs. No difference was found between pre- and postsurgery groups. P3d amplitudes showed no significant differences among the three groups. | Presurgery patients exhibited inhibition dysfunction, likely due to nerve tissue damage or brain structure alterations caused by tumor pressure and abnormal hormone levels. |
| Cao (2021) | Retrospective | PRLoma: 40  GHoma: 10 | 27 | EEG | To identify the electrophysiological changes related to attention processing in PA patients before and after treatment. | Preoperative patients had higher P200 amplitudes compared to HCs and postoperative patients. Postoperative patients' P200 amplitudes decreased to levels similar to those of HCs. | The P200 component can diagnose attention processing in preoperative PA patients and demonstrate improvement in postoperative patients. |
| Yuan  (2021) | Retrospective | GHoma: 29  NFPA: 31 | n/a | MRI | To investigate microstructural pathology in the cortex and white matter in GHoma patients. | GHoma had significantly increased cortical thickness throughout the bilateral cortex compared to NFPA patients. Microstructural pathology in the cortex and white matter were associated with neuropsychological dysfunction in GHoma patients. | Long-term persistent and excess serum GH/ IGF-1 levels alter the microstructure in the cortex and white matter in GHomas, which may be responsible for neuropsychological dysfunction. |
| Castinetti (2021) | Retrospective | PA: 64 | n/a | GB, SCWT, TMT, PASA, | To determine the long-term cognitive side effects of GK treatment in PA patients. | While up to 23.8% of the patients of the whole cohort presented at least one abnormal cognitive test, there was no significant difference in neurocognitive function between GK patients and non-GK patients. | While GK exposes patients to a well-known risk of pituitary deficiency, it does not seem to induce long-term cognitive consequences. |
| Hou (2021) | Retrospective | ACTHoma: 50 | 36 | MRI | To assess the reversibility of whole brain changes in remitted ACTHomas after TSS, and its correlations with clinical and hormonal parameters. | Widespread brain volume loss was observed in active ACTHomas compared to HCs. After TSS, all affected brain regions improved significantly. In patients with remitted ACTHomas, total gray matter and most brain regions fully recovered, showing volumes comparable to those of HCs. | The combination of fully and partially recovered brain areas after TSS aligns with the incomplete recovery of memory and cognitive function observed in ACTHoma patients in clinical practice. |
| Bala  (2022) | Retrospective | PRLoma: 27 | 27 | CTT, d2, TEA, DGS, DSST | To assess the attention processes and working memory in PRLoma patients. | Patients with PRLomas showed impairments in processing speed, working memory and attentional switching but not sustained attention. | Patients with PRLomas suffered from impaired cognitive functions, including attention and working memory. |
| Hatipoglu (2022) | Retrospective | GHoma: 33 | 30 | ANT, SVFT, KAS test, LAST, CDT, CT, JLOT, FRT, BNT, VMPT | To evaluate the relationship between mood status and cognitive functions with neurosteroid levels in patients with GHomas. | In GHoma patients, the time to complete the 1-second series was significantly higher, while scores on the SVFT, BNT, and the highest learning point of the VMPT were significantly lower compared to HCs. | Cognitive changes may occur in acromegaly, and neurosteroids may contribute to alterations in certain cognitive functions. |
| Chen  (2022) | Retrospective | PRLoma: 27 | 26 | EEG | To identify potential cognitive impairment in patients with PRLomas. | Patients with PRLomas elicited reduced P2, enhanced N2 and lower P3 amplitudes. | There are certain cognitive impairments in PRLoma patients; this may be attributed to the elevation of prolactin level. |
| Xie  (2022) | Retrospective | GHoma: 55 | 70 | MRI | To investigate the link between cognitive function and acromegaly-related CMBs in GHomas. | CMBs occurred in 29.1% of cases, with a significantly higher prevalence in acromegaly patients compared to the HCs. Binary logistic regression, adjusted for age, education, identified CMBs as an independent risk factor for cognitive impairment in acromegaly patients. | Increasing CMBs contribute to cognitive decline in patients with acromegaly. |
| Keil (2022) | Prospective | ACTH: 41 | n/a | WRAT, TMT, CVLT, | To investigate the effect of hypercortisolism on the developing brain, we conducted clinical, cognitive, and psychological evaluations of children with ACTHomas at diagnosis and one year after remission. | Comprehensive cognitive evaluations at baseline and one-year post-cure revealed a significant decline, primarily in nonverbal skills. Decrements were noted across various cognitive indices, with younger age and early pubertal stage contributing significantly to this decline. | Chronic glucocorticoid excess and subsequent eucortisolemia impair cognitive function in the developing brain. Younger children and those in early puberty are more vulnerable, while older adolescents show cognitive issues similar to adults with ACTHomas. |
| García-Casares (2020) | Retrospective | GHoma: 23 | n/a | MRI, MMSE, SCWT, TMT, DGS, DSST, VF, WMS | To assess the cognitive function, depression, and QoL in GHoma patients treated with pegvisomant compared to those treated with SRLs, and to examine the impact of these treatments on cognition and potential structural brain changes. | There is no significant differences between the groups in neuropsychological tests, depression, or quality of life, nor in whole-brain cortical thickness. In the SRL group, thalamus volume positively correlated with executive function, a correlation that was not observed in the pegvisomant group. | The type of pharmacological treatment in patients with GHomas and good glycemic control did not affect cognitive function or cortical brain thickness. |
| Wang (2023) | Prosepctive | GHoma: 27  NFPA: 29 | 24 | MMSE, MoCA, TMT, DGS, FAB | To assess the cognitive and emotional functions in individuals with GHomas and examine the impact of surgical treatment. | Compared to the HCs, patients with GHomas demonstrated poorer performance in memory and anxiety assessments. | Patients with GHomas had specific cognitive deficits and abnormal moods, potentially due to the overproduction of growth hormone. |
| Wisdom (2023) | Retrospective | NFPA: 229 | n/a | CFQ | To explore the links among patient factors, clinical information, and patient-reported quality of life in individuals with NFPAs. | Patients with hypopituitarism experienced lower energy levels, higher levels of fatigue, and decreased cognitive abilities. Among those who underwent radiation therapy, there were notably poorer reports of general health, physical well-being, physical fatigue, and cognitive performance. | Hypopituitarism, radiation therapy, and financial stressors are linked to a significantly reduced QoL in patients with NFPAs. |
| de Villiers  (2024) | Retrospective | NFPA: 18 | 19 | BTACT | To investigate whether sleep disturbances in patients with NFPAs contribute to their cognitive impairment. | NFPA patients have poorer cognitive performance compared to the HCs; however, these differences did not achieve statistical significance after applying the Bonferroni correction. | NFPA patients have relatively intact cognition and sleep quality, and sleep disruption does not mediate cognitive dysfunction. |

Abbreviation: DGS, Digit Span; DSST, Digit Symbol Substitution Test; AVLT, Rey Auditory Verbal Learning Test; LMW, Luria’s Memory Words Test; SCWT, Stroop Color-Word Test; TMT, Trail-Making Test; LCT, Letter Cancellation Tasks; TH, Tower of Hanoi; FACT-Cog, Functional Assessment of Cancer Therapy Cognitive Scale; ROCFT, Rey-Osterrieth Complex Figure Test; COWAT, Controlled Oral Word Association Test WMS, Wechsler Memory Scale; WRMT, Warrington Recognition Memory test; Corsi, Corsi Block-Tapping Test; RCPM, Raven’s Colored Progressive Matrices; RMTF, Recognition Memory Test of Faces; BD, Block Design; WAIS, Wechsler Adult Intelligence Scale; RMT, Recognition Memory Test; CMVM, Cronholm –Molander Verbal Memory Test ; AM, Austin Maze; APT RT-2, APT Two-way Reaction Time test; APT RT-inhibition, APT Inhibition test; WRAT, Wide Range Achievement Test; MRI, Magnetic Resonance Imaging; CT, Computed Tomography; RFC, Rey Figure Copy; SCOLP, Speed and Capacity of Language Processing; CVLT, California Verbal Learning Test; VOSP, Visual Object and Spatial Perception; AMIPB, Adult Memory and Information Processing Battery; TEA, Test of Everyday Attention; BSRT, Buschke Selective Reminding Test; CFQ, Cognitive Failures Questionnaire; MWT, Multiple Choice Word Fluency Test; EEG, Electroencephalography; THS, Tower of Hanoi-Sevilla; IST-2000, Intelligence Structure Test-2000; d2, d2-Letter Cancellation test; MMSE, Mini Mental State Examination; LDST, Letter-Digit Substitution Test; Digit-Deletion Test, DDT; GIT-2, Groninger Intelligence Test-2; RFFT, Ruff Figural Fluency Test; WS, Word Span; ANT, Attention Network Test; TAP, Testbatterie zur Aufmerksamkeitspru ̈fung; AKT, AltersKonzentrationstest; SPM, Raven Standard Progressive Matrices Test; RWT, Regensburger Wortflu ̈ssigkeits-Test; IGT, Iowa Gambling Task; BVRT, Benton Visual Retention Test; WCST, Wisconsin Card Sorting Test; BT, Barcelona Test; IPQ-R, The revised Illness Perception Questionnaire; VLMT, Verbal Learning and Memory Test; RWT, Regensburger Verbal Fluency Test; HSCT, Hayling Sentence Completion Test; SART, Sustained Atten- tion to Response Task; CAMCOG, Cambridge Cognitive Examination; MCCB, MATRICS Consensus Cognitive Battery; BACSSC, Brief Assessment of Cognition in Schizophrenia-Symbol Coding; CFAN, Category Fluency-Animal Naming test; CPTIP, Continuous Performance Test-Identical Pairs; SS, Spatial Span; UMLNS, University of Maryland Letter-Number Span; HVLT-R, Hopkins Verbal Learning Test-Revised; BVMT-R, Brief Visuospatial Memory Test-Revised; NAB-M, Neuropsychological Assessment Battery- Mazes; MoCA, Montreal Cognitive Assessment; SSRT, Short Story Recall Test; FAB, Frontal Assessment Battery; RCPM, Raven’s Coloured Progressive Matrices; NART, National Adult Reading Test; SRMS, Self-rating Memory Scale; RMT, Recognition Memory Test; GB, Grober and Buschke test; PASA, Paced Auditory Serial Attention test; CTT, Color Trails Test; ANT, Animal Naming Test; SVFT, Switching Verbal Fluency Test; LAST, Luria’s Alternating Series Tests; CDT, Clock Drawing Test; CT, Construction Test; JLOT, Judgement of Line Orientation Test; FRT, Facial Recognition Test; BNT, Boston Naming Test Short Form; VMPT, Oktem Verbal Memory Processes Test; BTACT, Brief Test of Adult Cognition by Telephone; NFPA, non-functional pituitary adenoma; QoL, quality of life; RT, radiotherapy; GHom, growth hormone-secreting adenoma; SRL, somatostatin analogue; ACTHoma, adrenocorticotropic hormone-secreting adenoma; CMB, cerebral micro-bleeding; PRLoma, prolactin-secreting adenoma; TSS, transsphenoidal surgery; HC, healthy control; GKRS, gamma-knife radiosurgery; PA, pituitary adenoma; IGF-1, insulin-like factor-1; EEG, electroencephalography; GA, 18 beta-glycyrrhetinic acid; EMMN, expression-related mismatch negativity; VF, verbal fluency; RSFC, resting-state functional connectivity; RIBD, radiation-induced brain damage
