# Supplementary material for: The impact of pituitary adenomas on cognitive performance: a systematic review
Source: Front Endocrinol (Lausanne). 2025 Apr 30;16:1534635. doi: 10.3389/fendo.2025.1534635 (PMC12074915; doi:10.3389/fendo.2025.1534635)
Supplement: Supplementary file 3 [file Table3.docx]

**Supplement 3.** Summary of risk of bias assessment for the included studies, evaluating selection, detection, and analysis bias based on predefined criteria. Each study was classified as having low, high, or unclear risk in these domains.

|  |  | Analysis Bias | Detection Bias | Selection Bias: age | Selection Bias: gender | Selection Bias: intellectual ability |  |  |  | Analysis Bias | Detection Bias | Selection Bias: age | Selection Bias: gender | Selection Bias: intellectual ability |  |  |
| --- | --- | --- | --- | --- | --- | --- | --- | --- | --- | --- | --- | --- | --- | --- | --- | --- |
| de Villiers | 2024 | ⚫ | ⚫ | ⚫ | ⚫ | ⚫ |  | Brummelman | 2015 | ⚫ | ⚫ | ⚫ | ⚫ | ⚫ |  |  |
| Wang | 2023 | ⚫ | ⚫ | ⚫ | ⚫ | ⚫ |  | Lecumberri | 2015 | ⚫ | ⚫ | ⚫ | ⚫ | ⚫ |  |  |
| Wisdom | 2023 | ⚫ | ⚫ | ⚫ | ⚫ | ⚫ |  | Crespo | 2015 | ⚫ | ⚫ | ⚫ | ⚫ | ⚫ |  |  |
| García-Casares | 2023 | ⚫ | ⚫ | ⚫ | ⚫ | ⚫ |  | Bas-Hoogendam | 2015 | ⚫ | ⚫ | ⚫ | ⚫ | ⚫ |  |  |
| Bala | 2022 | ⚫ | ⚫ | ⚫ | ⚫ | ⚫ |  | Tiemensma | 2015 | ⚫ | ⚫ | ⚫ | ⚫ | ⚫ |  |  |
| Chen | 2022 | ⚫ | ⚫ | ⚫ | ⚫ | ⚫ |  | Andela | 2013 | ⚫ | ⚫ | ⚫ | ⚫ | ⚫ |  |  |
| Xie | 2022 | ⚫ | ⚫ | ⚫ | ⚫ | ⚫ |  | Yedinak | 2013 | ⚫ | ⚫ | ⚫ | ⚫ | ⚫ |  |  |
| Hatipoglu | 2022 | ⚫ | ⚫ | ⚫ | ⚫ | ⚫ |  | Martín-Rodríguez | 2013 | ⚫ | ⚫ | ⚫ | ⚫ | ⚫ |  |  |
| Keil | 2022 | ⚫ | ⚫ | ⚫ | ⚫ | ⚫ |  | O. Ragnarsson | 2012 | ⚫ | ⚫ | ⚫ | ⚫ | ⚫ |  |  |
| Yuan | 2021 | ⚫ | ⚫ | ⚫ | ⚫ | ⚫ |  | Brummelman | 2012 | ⚫ | ⚫ | ⚫ | ⚫ | ⚫ |  |  |
| Cao | 2021 | ⚫ | ⚫ | ⚫ | ⚫ | ⚫ |  | Brummelman | 2012 | ⚫ | ⚫ | ⚫ | ⚫ | ⚫ |  |  |
| Cao | 2021 | ⚫ | ⚫ | ⚫ | ⚫ | ⚫ |  | Sievers | 2012 | ⚫ | ⚫ | ⚫ | ⚫ | ⚫ |  |  |
| Castinetti | 2021 | ⚫ | ⚫ | ⚫ | ⚫ | ⚫ |  | Petry | 2011 | ⚫ | ⚫ | ⚫ | ⚫ | ⚫ |  |  |
| Hou | 2021 | ⚫ | ⚫ | ⚫ | ⚫ | ⚫ |  | Müssig | 2011 | ⚫ | ⚫ | ⚫ | ⚫ | ⚫ |  |  |
| Cao | 2020 | ⚫ | ⚫ | ⚫ | ⚫ | ⚫ |  | Brummelman | 2011 | ⚫ | ⚫ | ⚫ | ⚫ | ⚫ |  |  |
| Marsh | 2020 | ⚫ | ⚫ | ⚫ | ⚫ | ⚫ |  | Psaras | 2011 | ⚫ | ⚫ | ⚫ | ⚫ | ⚫ |  |  |
| Song | 2020 | ⚫ | ⚫ | ⚫ | ⚫ | ⚫ |  | Tiemensma | 2010 | ⚫ | ⚫ | ⚫ | ⚫ | ⚫ |  |  |
| Kan | 2019 | ⚫ | ⚫ | ⚫ | ⚫ | ⚫ |  | Leon-Carrion | 2010 | ⚫ | ⚫ | ⚫ | ⚫ | ⚫ |  |  |
| Butterbrod | 2019 | ⚫ | ⚫ | ⚫ | ⚫ | ⚫ |  | Tiemensma | 2010 | ⚫ | ⚫ | ⚫ | ⚫ | ⚫ |  |  |
| Crouzeix | 2019 | ⚫ | ⚫ | ⚫ | ⚫ | ⚫ |  | Psara | 2010 | ⚫ | ⚫ | ⚫ | ⚫ | ⚫ |  |  |
| Zarino | 2019 | ⚫ | ⚫ | ⚫ | ⚫ | ⚫ |  | Müssig | 2009 | ⚫ | ⚫ | ⚫ | ⚫ | ⚫ |  |  |
| Wennberg | 2019 | ⚫ | ⚫ | ⚫ | ⚫ | ⚫ |  | Tanriverdi | 2009 | ⚫ | ⚫ | ⚫ | ⚫ | ⚫ |  |  |
| Song | 2018 | ⚫ | ⚫ | ⚫ | ⚫ | ⚫ |  | van Beek | 2007 | ⚫ | ⚫ | ⚫ | ⚫ | ⚫ |  |  |
| Montalvo | 2018 | ⚫ | ⚫ | ⚫ | ⚫ | ⚫ |  | Hook | 2007 | ⚫ | ⚫ | ⚫ | ⚫ | ⚫ |  |  |
| Tooze | 2018 | ⚫ | ⚫ | ⚫ | ⚫ | ⚫ |  | Heald | 2006 | ⚫ | ⚫ | ⚫ | ⚫ | ⚫ |  |  |
| Wang | 2018 | ⚫ | ⚫ | ⚫ | ⚫ | ⚫ |  | Bhansali | 2004 | ⚫ | ⚫ | ⚫ | ⚫ | ⚫ |  |  |
| Solomon | 2018 | ⚫ | ⚫ | ⚫ | ⚫ | ⚫ |  | Noad | 2004 | ⚫ | ⚫ | ⚫ | ⚫ | ⚫ |  |  |
| Cao | 2017 | ⚫ | ⚫ | ⚫ | ⚫ | ⚫ |  | Bellhouse | 2003 | ⚫ | ⚫ | ⚫ | ⚫ | ⚫ |  |  |
| Alibas | 2017 | ⚫ | ⚫ | ⚫ | ⚫ | ⚫ |  | Bülow | 2002 | ⚫ | ⚫ | ⚫ | ⚫ | ⚫ |  |  |
| Hendrix | 2017 | ⚫ | ⚫ | ⚫ | ⚫ | ⚫ |  | Starkman | 2001 | ⚫ | ⚫ | ⚫ | ⚫ | ⚫ |  |  |
| Wang | 2017 | ⚫ | ⚫ | ⚫ | ⚫ | ⚫ |  | Guinan | 1998 | ⚫ | ⚫ | ⚫ | ⚫ | ⚫ |  |  |
| Yao | 2017 | ⚫ | ⚫ | ⚫ | ⚫ | ⚫ |  | Peace | 1998 | ⚫ | ⚫ | ⚫ | ⚫ | ⚫ |  |  |
| Shan | 2017 | ⚫ | ⚫ | ⚫ | ⚫ | ⚫ |  | Peace | 1997 | ⚫ | ⚫ | ⚫ | ⚫ | ⚫ |  |  |
| Bala | 2016 | ⚫ | ⚫ | ⚫ | ⚫ | ⚫ |  | Mauri | 1993 | ⚫ | ⚫ | ⚫ | ⚫ | ⚫ |  |  |
| van der Werff | 2015 | ⚫ | ⚫ | ⚫ | ⚫ | ⚫ |  | Grattan-Smith | 1992 | ⚫ | ⚫ | ⚫ | ⚫ | ⚫ |  |  |

Green dot = low risk; Red dot = high risk; Yellow dot = unclear risk
