# Supplementary material for: The impact of pituitary adenomas on cognitive performance: a systematic review
Source: Front Endocrinol (Lausanne). 2025 Apr 30;16:1534635. doi: 10.3389/fendo.2025.1534635 (PMC12074915; doi:10.3389/fendo.2025.1534635)
Supplement: Supplementary file 4 [file Table4.docx]

| **Outcome** | **Studies (n)** | **Percentage (%)** |
| --- | --- | --- |
| Studies reporting psychiatric outcomes | 49 |  |
| - Depression | 11 | 22.4 |
| - Anxiety | 10 | 20.4 |
| - Other psychiatric symptoms (including apathy, irritability, social phobia) | 6 | 12.2 |
| - No association between psychiatric disorders and cognitive impairment | 10 | 20.4 |
| Studies reporting quality of life | 9 |  |
| - Decreased QoL in PA patients | 5 | 55.6 |
| - Negative impact of radiotherapy on QoL | 2 | 22.2 |
| - Improved QoL after surgery | 1 | 11.1 |

**Supplement 4.** Summary of psychiatric and QoL outcomes in PA patients
